# Supplementary material for: Development of mirror-image monobodies targeting the oncogenic BCR::ABL1 kinase
Source: Nat Commun. 2024 Dec 23;15:10724. doi: 10.1038/s41467-024-54901-y (PMC11666773; doi:10.1038/s41467-024-54901-y)
Supplement: Supplementary file 1 — Supplementary Information [file 41467_2024_54901_MOESM1_ESM.pdf]

Supplementary Information

Development of mirror-image monobodies targeting the oncogenic BCR::ABL1 kinase

Nina Schmidt[1], Amit Kumar[1], Lukas Korf[2], Adrian Valentin Dinh-Fricke[1], Frank Abendroth[3], Akiko Koide[4,5], Uwe Linne[6], Magdalena Rakwalska-Bange[1], Shohei Koide[5,7], Lars-Oliver Essen[2], Olalla Vázquez[3,8,*], Oliver Hantschel[1,*]

[1] Institute of Physiological Chemistry, Faculty of Medicine, Philipps University of Marburg, 35032 Marburg, Germany

[2] Faculty of Chemistry and Unit for Structural Biology, Philipps University of Marburg, 35032 Marburg, Germany

[3] Faculty of Chemistry and Unit for Chemical Biology, Philipps University of Marburg, 35032 Marburg, Germany

[4] Department of Medicine, New York University School of Medicine, 522 1st Avenue, New York, NY 10016, USA

[5] Laura and Isaac Perlmutter Cancer Center, New York University Langone Health, 522 1st Avenue, New York, NY 10016, USA

[6] Faculty of Chemistry and Unit for Mass Spectrometry, Philipps University of Marburg, 35032 Marburg, Germany

[7] Department of Biochemistry and Molecular Pharmacology, New York University School of Medicine, 522 1st Avenue, New York, NY 10016, USA

[8] Center for Synthetic Microbiology (SYNMIKRO), Philipps University of Marburg, 35032 Marburg, Germany

[*] Correspondence should be addressed to O.H. (oliver.hantschel@uni-marburg.de) and O.V. (olalla.vazquez@staff.uni-marburg.de)

Content:

Supplementary Figures S1-S23

Supplementary Tables S1-S10

Table of Contents

Supplementary Note 1: Abbreviations, materials and general procedures 3

Supplementary Note 2: Monobody Selection with D-BCR::ABL1 SH2 7

Supplementary Note 3: Fluorescence polarization assay of L-BCR::ABL1 SH2 with a L-pY peptide 8

Supplementary Note 4: Isothermal titration calorimetry (ITC) data of DAM26/28/30.1/30.2 9

Supplementary Note 5: Synthesis of the Bcr-Abl SH2 A198C domain for protein co-crystallization 10

Synthesis of N-terminal Bcr-Abl SH2 peptide in D-configuration (D-1) 11

Synthesis of C-terminal Bcr-Abl SH2 peptide in D-configuration (D-2) 12

Synthesis of ligated Bcr-Abl SH2 A198C in D-configuration (D-3) 13

Supplementary Note 6: ITCs of L-AS25 and L-pY peptide binding to recombinant L-Abl SH2 A198C 14

Supplementary Note 7: Supplementary figures of the heterochiral Abl SH2-monobody complexes 15

Supplementary Note 8: Circular dichroism spectroscopy measurement of rec. L-DAM21 D83A/E85A 16

Supplementary Note 9: Synthesis of monobodies DAM27 and DAM21 17

Synthesis of the N-terminal DAM27 peptide in L-configuration (L-4) 18

Synthesis of the N-terminal DAM27 peptide in D-configuration (D-4) 19

Synthesis of the C-terminal DAM27 peptide in L-configuration (L-5) 20

Synthesis of the C-terminal DAM27 peptide in D-configuration (D-5) 21

Synthesis of ligated DAM27 peptide A61C in L-configuration (L-6) 22

Synthesis of ligated DAM27 peptide A61C in D-configuration (D-6) 23

Desulfurization of the DAM27 peptide in L-configuration (L-7) 24

Desulfurization of the DAM27 peptide in D-configuration (D-7) 25

Synthesis of the N-terminal DAM21 peptide in L-configuration (L-8) 26

Synthesis of the N-terminal DAM21 peptide in D-configuration (D-8) 27

Synthesis of the C-terminal DAM21 peptide in L-configuration (L-9) 28

Synthesis of the C-terminal DAM21 peptide in D-configuration (D-9) 29

Supplementary Note 10: Secondary structure content of synthetic monobodies 30

Supplementary Note 11: Unfolding and Refolding of DAM21/27 measured by nanoDSF 31

Supplementary Note 12: ITC data of rec. L-monobodies DAM21/27 with rec. L-Abl SH2 32

Supplementary Note 13: Sequence alignment of βC/βD strands of SH2 domains 33

Supplementary Note 14: Pulldown of BCR::ABL1 from K562 cells with D-DAM21/27 34

Supplementary Note 15: Binding of monobodies to BCR::ABL1 in permeabilized K562 cells 36

Supplementary References 37

Supplementary Note 1:

Abbreviations, materials and general procedures

Abbreviations:

Ac_2O	acetic anhydride
AIM	auto induction medium
BCR::ABL1	fusion product of breakpoint cluster region and Abelson tyrosine kinase
CD	circular dichroism
DCE	1,2-dichloroethane
DCM	dichloromethane
DIC	*N*,*N*-diisopropylcarbodiimide
DIPEA	*N*,*N*-diisopropylethylamine
DMF	*N*,*N*-dimethylformamide
DTT	dithiothreitol
EDT	1,2-ethanedithiol
EDTA	ethylenediaminetetraacetic acid
ESI-MS	electrospray ionization mass spectrometry
Et_2O	diethyl ether
FAM	5-carboxyfluoresceine
Fmoc	9-fluorenylmethoxycarbonyl
HATU	1-[bis(dimethylamino)methylene]-1H-1,2,3-triazolo[4,5-b]pyridinium 3-oxide
HBTU	3-[bis(dimethylamino)methyliumyl]-3H-benzotriazol-1-oxide
HEPES	4-(2-hydroxyethyl)-1-piperazineethanesulfonic acid
HR-MS	high-resolution mass spectrometry
IPTG	isopropyl β-D-1-thiogalactopyranoside
ITC	isothermal titration calorimetry
LB	lysogeny broth
m/z	mass-to-charge ratio
MeCN	acetonitrile
MeDbz	3-amino-4-(methylamino)-benzoic acid (Dawson linker)
MeNbz	*N*-acyl-*N′*-methylacylurea
MESNa	2-mercaptoethane sulfonate sodium salt
MPAA	4-mercaptophenyl acetic acid
MRE	mean residue ellipticity

MS-ESI$^+$	electrospray ionization mass spectrometry in positive ion mode
nanoDSF	nano differential scanning fluorimetry
NCL	native chemical ligation
Ni-NTA	nickel nitrilotriacetic acid
NMM	*N*-methyl morpholine
NMP	*N*-methyl pyrrolidone
PBS	phosphate buffered saline
PEC	Peptide Easy Clean kit
PyBOP	benzotriazol-1-yloxytripyrrolidinophosphonium
RP-HPLC	reverse phase high performance liquid chromatography
rpm	revolutions per minute
RT	room temperature
SEC	size exclusion chromatography
SPPS	solid phase peptide synthesis
TB	terrific broth
TCEP	tris(2-carboxyethyl)phosphine
TEV	tobacco etch virus
TFA	trifluoroacetic acid
TIS	triisopropylsilane
Tris	tris(hydroxymethyl)aminomethane

Materials: All commercially purchased reagents were used without further purification as delivered from the corresponding company. The respective reagents were purchased from the following companies: guanidine-HCl biochemical grade, NaCl, Na_2HPO_4 x 2 H_2O, L-glutathione, HEPES, Tris, imidazole, DIPEA, 2,6-lutidine, pyridine, NMM, DIC, piperidine and LC-MS grade TFA from Carl Roth (Germany); TentaGel S RAM resin, NMP and Fmoc-MeDbz-OH from Iris Biotech (Germany); Fmoc-protected L-amino acids, HBTU and OxymaPure from Carbolution (Germany); VA-044 x 2HCl, MESNa and TCEP-HCl from TCI (Germany); 4-nitrophenyl chloroformate and TFA for peptide cleavage from Acros Organics (USA); D-biotin from J&K Scientific (Germany); Fmoc-protected D-amino acids from Merck (Germany) or Carbolution (Germany); *N*-acetoxy succinimide from ChemPur (Germany); MeCN as HPLC LC-MS grade, DMF anhydrous and 1,2-dichloro ethane from VWR (France); Fmoc-Gly-Ser(psiMe,Mepro)-OH, MPAA, EDT, TIS, DEE, HPLC-grade DCM, kanamycin sulfate, IPTG and LB broth from Sigma-Aldrich (Germany); Gibco 10x PBS and Slide-A-Lyzer 3.5K Dialysis Cassettes G2 (#87722, #87723, #87724) from Thermo Fisher Scientific (USA); formic acid optima from Fisher Chemical (USA); EDTA-free protease inhibitor tablets and DNase I from Roche (Switzerland); HATU and PyBOP from BLDpharm (Germany); microscale columns with PTFE filter (#35.091) from CEM (USA). DMF was employed as peptide grade (Iris Biotech, Germany). The FAM-labeled L- and D-pYEEI peptides for FP assay and ITC measurements were ordered from Peptide Synthetics (UK). Water was purified with a Milli-Q Ultra-Pure (TKA, Germany) or Milli-Q Advantage A10 (Merck Millipore, Germany) Water Purification System.

Solid phase peptide synthesis (SPPS): Both L- and D-peptides were prepared with the same protocol as described below by automated synthesis using the Fmoc solid phase strategy, unless noted otherwise. The synthesis was performed using microscale columns with PTFE filter (CEM) in a ResPep SLi (Intavis) parallel synthesizer. TentaGel S RAM resin (0.22 mmol/g) was used for synthesis. The reagent amounts correspond to 5 µmol scale synthesis.

Manual coupling of Fmoc-MeDbz-OH: The corresponding amount of resin for a 5 µmol scale synthesis (~23 mg) was weighed in microscale columns and 200 µL DMF were added. The resin was swelled for 30 min. DMF/piperidine (4:1, 200 µL) was added to the resin. After 5 min, the procedure was repeated once. The resin was washed (6 × 200 µL DMF) and 200 µL of a 0.25 M solution of Fmoc-MeDbz-OH (10 eq.), 0.25 M HBTU (10 eq.) and 0.5 M DIPEA (20 eq.) was added to the resin. The procedure was repeated after 30 min and the resin was washed (6 × 200 µL DMF).

Automated washing of the resin: The resin was washed (4 × 300 µL DMF) prior to synthesis start by the device.

Automated deprotection of the Fmoc group: DMF/piperidine (4:1, 150 µL) was added to the resin. After 5 min, the procedure was repeated once. The resin was washed (1 × 300 µL DMF, 3 × 225 µL DMF).

Automated coupling of amino acid monomers: Standard amino acids were coupled by charging the reactor with 53 µL of a solution of the corresponding Fmoc-amino acid (0.5 M solution in NMP, 5 eq.), 15 µL OxymaPure (6 eq.), 13 µL DIC (5 eq.) and 29 µL NMP. The resulting solution was incubated for 20 min at 50 °C, the resin was filtered and subjected to a recoupling step following the same procedure. Fmoc-Gly-(L- or D-)-Ser(psiMe,Mepro)-OH was coupled by charging the reactor with a solution of the corresponding Fmoc-pseudoproline (0.5 M solution in NMP, 5 eq.), HATU (4.8 eq.) and NMM (13 eq.). The resulting solution was incubated for 30 min, the resin was filtered and subjected to a recoupling step following the same procedure.

Automated capping: 0.5 M *N*-acetoxy succinimide in pyridine/DMF (1:6, 120 µL) was added for 8 min. Then, the resin was washed (3 × 225 µL DMF). The capping reagent was exchanged for a fresh-prepared solution every 48 h.

Automated final Fmoc deprotection and washing of resin: DMF/piperidine (4:1, 150 µL) was added to the resin. After 5 min, the procedure was repeated twice. The resin was finally washed (4 × 300 µL DMF, 4 × 150 µL ethanol, 5 x 150 µL DCM).

Manual coupling of D-biotin after completion of synthesis: 500 µL of a 0.1 M solution of D-biotin (10 eq.), 0.1 M PyBOP (10 eq.) and 0.2 M NMM (20 eq.) were added to the resin. After 30 min, the procedure was repeated once with 500 µL of a 0.1 M solution of D-biotin (10 eq.), 0.1 M HATU (10 eq.) and 0.2 M NMM (20 eq.). Then, the resin was washed (6 × 200 µL DMF, 6 × 200 µL DCM).

Final cleavage: The resin was treated with the cleavage cocktail (92.5% TFA, 2.5% H_2O, 2.5% TIS, 2.5% EDT up to 2 mL of cocktail for ~30 mg of resin). The resulting suspension was shaken for 2.5 h and the resin was filtered off. The resulting filtrated solution was concentrated with nitrogen gas until less than 1 mL of cleavage cocktail was left, and added to ice-cold Et_2O (10 mL of Et_2O for each 1 mL of TFA). After 10 min, the mixture was centrifuged (8000 rpm, 10 min, 4 °C), decantated, and the solid was again suspended in 10 mL of fresh ice-cold Et_2O, sonicated and centrifuged again. This procedure was repeated once and the solid residue was dried. Each peptide was dissolved as indicated in sections 3 and 6 and purified by semipreparative reverse-phase (RP)-HPLC or the Peptide Easy Clean (PEC) kit (see sections 6.11-6.12).

Purification: The peptides were purified by semipreparative HPLC, performed on a Thermo Scientific Dionex UltiMate 3000 series instrument (column: *Kinetex EVO C18* 5 µm, 150 × 21.2 mm, pore size 100 Å, flow rate of 20 mL/min) using eluents A (99.9% H_2O, 0.1% TFA) and B (99.9% acetonitrile, 0.1% TFA) in the corresponding linear gradient as written for each peptide (sections 3 and 6). Detection of the signals was achieved with a UV-detector at wavelength 220 nm. The collected fractions were lyophilized and stored at -20 °C.

Characterization of probes: Analytical HPLC chromatograms were performed on three different systems:

a) An Agilent 1260 infinity II LC system equipped with a 6120B Single Quadrupole mass spectrometer (column: *ZORBAX Eclipse XDB-C18* 5 µm, 150 × 4.6 mm, pore size 80 Å). A flow rate of 1 mL/min and a column temperature of 55 °C were used. Detection of signals were achieved with a UV detector at wavelength 220 nm. Electrospray Ionization Mass Spectrometry (ESI-MS) was performed by direct injection on the 6120B Single Quadrupole mass spectrometer in positive scan mode. The following method was used:

Tab. S1: HPLC method used on the Agilent LC system (a). Measurements of analytical data with this method are mentioned in the footnote of the corresponding chromatograms.

HPLC Method	Eluent A	Eluent B	Gradient
A	99.95% H_2O, 0.05% TFA	99.97% acetonitrile, 0.03% TFA	5 – 75% B in 25 min

b) The UHPLC system was the Thermo Scientific Dionex UltiMate 3000 RS instrument (column: *Kinetex EVO C18* 1.7 µm, 50 × 2.1 mm, pore size 100 Å). A flow rate of 0.5 mL/min and a column temperature of 40 °C were used. Detection of signals was achieved with a UV detector at wavelength 220 nm. ESI-MS was performed by direct injection on a Thermo Scientific MSQ Plus mass spectrometer in positive scan mode. The following methods were used:

Tab. S2: HPLC method used on the Thermo Scientific LC system (b). Measurements of analytical data with these methods are mentioned in the footnote of the corresponding chromatograms.

HPLC Method	Eluent A	Eluent B	Gradient
B	99.95% H_2O, 0.05% TFA	99.97% acetonitrile, 0.03% TFA	5 – 30% B in 25 min
C	99.95% H_2O, 0.05% TFA	99.97% acetonitrile, 0.03% TFA	5 – 40% B in 25 min

c) High-resolution electrospray ionization mass spectra (HR-MS) were acquired at the Mass Spectrometry Facility of the Philipps-University Marburg with an LTQ-FT Ultra mass spectrometer (Thermo Fischer Scientific). The resolution was set to 100000.

Supplementary Note 2:

Monobody Selection with D-BCR::ABL1 SH2

Monobody selection was performed via phage and subsequent yeast display. First, four rounds of phage display were performed with phagemid containing the monobody library and the biotinylated target D-BCR::ABL1 SH2 immobilized on Streptavidin MagneSphere Paramagnetic Particles (Z5481, Promega). The first round is performed manually where the phage library with ~10^{11} phage particles is added to a final concentration of the target protein at 100 nM. After washing the beads four times with TBS, log-phase XL-1/pMCSG21 cells are added and incubated with hyper phage overnight. Rounds 2 – 4 are performed with 100, 50, and 50 nM of the target protein on a KingFisher 700 (Thermo Fisher Scientific) instrument with phage particles precipitated from the supernatant of the overnight culture using PEG/NaCl solution and incubation at 4 °C. The phage elution after each round of selection is incubated with log-phase XL-1/pMCSG21 cells overnight and used for the next round of selection.

For yeast selection, the DNA sequences of binding monobody variants were amplified by 5'-3'-loop-shuffling PCR using KOD polymerase and cleaned with the QIAquick PCR Purification Kit (Qiagen). The resulting DNA fragments are mixed with digested yeast display vector pGalAgaVHH-V5 (*Nco*I and *Xho*I) and transformed into EBY100 yeast cells via PEG3500/LiAc transfer.[1] Monobody expression was induced with galactose and cells were incubated with the target protein at 1000 (1st round), 500 (2nd round), 200 (3rd round) and 50 nM (4th round). Cells were stained with mouse anti-V5 and FITC-coupled anti-mouse IgG for monobody expression and with streptavidin-DyLight650 for the biotinylated target. Double-positive cells were visualized and sorted via FACS on a Sony Cell Sorter SH800S.

Binding monobodies were sequenced by standard Sanger sequencing, re-cloned into the pHFT2 expression vector, digested with BamHI and XhoI, for bacterial expression in *E. coli* by standard Gibson assembly (NEB). Prior to Gibson assembly, the monobody fragments were amplified from the isolated yeast plasmids via PCR using 5'-gtgaaaacctgtacttccagggatccatggctgcttcttctg-3' and 5'-cagtggtggtggtggtggtgctcgagctaggtacggtagttaatc-3' as primers.

Supplementary Note 3:

Fluorescence polarization assay of L-BCR::ABL1 SH2 with a L-pY peptide

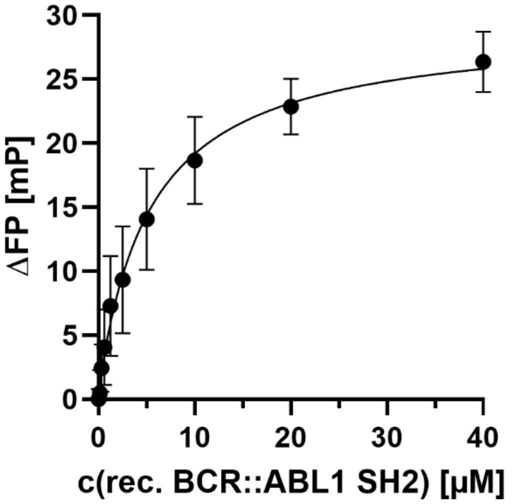

Fig. S1: Fluorescence polarization measurement of the recombinantly expressed BCR::ABL1 SH2 domain binding to a FAM-labelled L-pY peptide. The binding curve from three independent experiments was fitted via GraphPad Prism 8 based on a one site-specific binding model resulting in a K_d value of 4.89 µM. Based on this binding curve a concentration of BCR::ABL1 SH2 where the binding activity was 80 % was estimated at 10 µM and used for the competitive measurements with D-BCR::ABL1 and monobodies. Source data of this figure are provided as a Source Data file.

Supplementary Note 4:

Isothermal titration calorimetry (ITC) data of DAM26/28/30.1/30.2

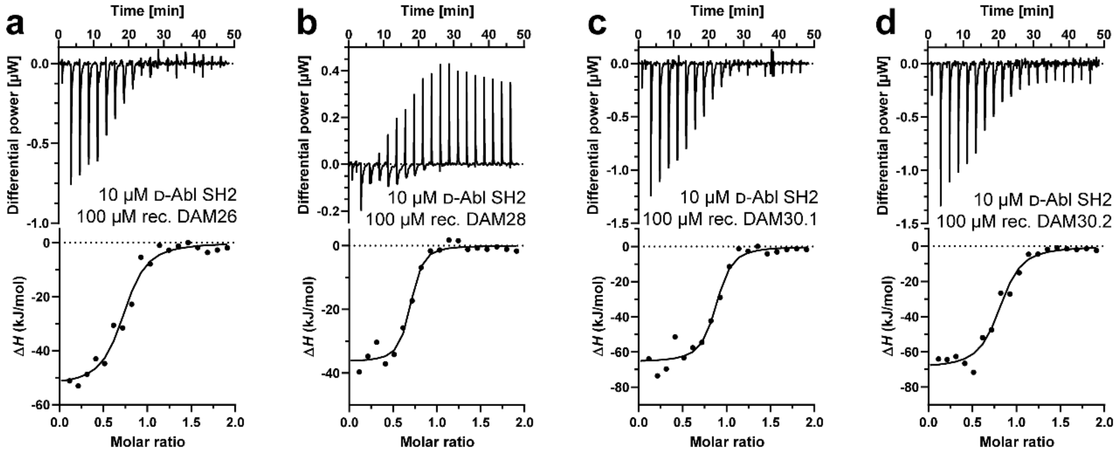

e

	DAM26
K_d [nM]	249 ± 112
N	0.709 ± 0.028
ΔH [kJ/mol]	-53.0 ± 3.81
ΔG [kJ/mol]	-37.7
$-T\Delta S$ [kJ/mol]	15.3

f

	DAM28
K_d [nM]	58.9 ± 27.9
N	0.660 ± 0.016
ΔH [kJ/mol]	-36.5 ± 1.71
ΔG [kJ/mol]	-41.3
$-T\Delta S$ [kJ/mol]	-4.78

g

	DAM30.1
K_d [nM]	90.3 ± 46.0
N	0.844 ± 0.023
ΔH [kJ/mol]	-65.7 ± 3.75
ΔG [kJ/mol]	-40.2
$-T\Delta S$ [kJ/mol]	25.5

h

	DAM30.2
K_d [nM]	189 ± 82.4
N	0.786 ± 0.026
ΔH [kJ/mol]	-69.2 ± 4.34
ΔG [kJ/mol]	-38.4
$-T\Delta S$ [kJ/mol]	30.8

Fig. S2: Binding of recombinantly expressed monobodies DAM26/28/30.1/30.2 to synthetic D-Abl SH2. (**a-d**) Isothermal titration calorimetry (ITC) measurements of recombinant L-monobodies (**a**) DAM26, (**b**) DAM28, (**c**) DAM30.1 and (**d**) DAM30.2 titrated to the synthetic D-Abl SH2 domain. Each panel shows the raw heat signal of an ITC experiment (top) and the integrated calorimetric data of the area of each peak (bottom). The continuous line represents the best fit of the data based on a 1:1 binding model computed from the MicroCal software. Binding parameters including K_d value, stoichiometry (N), enthalpy (ΔH), free enthalpy (ΔG) and $-T\Delta S$ calculated from the fit of each experiment are shown in (**e-h**) below the calorimetric data. A representative measurement of at least two ITC experiments for each monobody is shown.

Supplementary Note 5:

Synthesis of the Bcr-Abl SH2 A198C domain for protein co-crystallization

Tab. S3: Sequences of the synthesized peptide fragments. The underlined residues indicate the incorporation of the Fmoc-Gly-Ser(psiMe,Mepro)-OH pseudoproline.

Peptide	Sequence	Mass [Da]
**1**	Acetyl-KKKKKSG-VNSLEKHSWYHGPVSRNAAEYLLSSGINGSFLV-RESESSPGQRSISLRYEGRVYHYRINT-MeNbz-$CONH_2$	7856.8
**2**	H_2N-CSDGKLYVSSESRFNTLAELVHHHSTVADGLITTLHYPAPKR-NK-$CONH_2$	4893.5
**3**	Acetyl-KKKKKSG-VNSLEKHSWYHGPVSRNAAEYLLSSGINGSFLV-RESESSPGQRSISLRYEGRVYHYRINTCSDGKLYVSSESRFNTLAEL-VHHHSTVADGLITTLHYPAPKRNK-$CONH_2$	12559.1

Synthesis of N-terminal Bcr-Abl SH2 peptide in D-configuration (D-1)

Peptide **D-1** was synthesized with the usage of the D-pseudoproline Fmoc-Gly-D-Ser(psiMe,Mepro)-OH and final *N*-terminal capping. The MeDbz linker activation was performed on resin, which was treated with 4-nitrophenyl chloroformate (2 eq., 20 mM) dissolved in 2500 µL 1,2-dichloroethane (DCE) for 45 min. The reaction was repeated twice. After washing the resin (6 × DCE, 6 × anhydrous DMF), 2500 µL of DIPEA (50 eq., 0.5 M) in anhydrous DMF were added. The reaction proceeded for 15 min, repeated for 30 min and then conducted a third time for 15 min. After cleavage of the peptide from the resin, the precipitated peptide was dissolved in H_2O/MeCN (50:50, v/v) with addition of 0.1% TFA and purified via semipreparative RP-HPLC using a linear gradient of 5-40% MeCN with 0.1% TFA over 30 min. After lyophilization, 30.3 mg (3.17 µmol, 13%, 25 µmol scale) of the desired peptide were obtained as TFA salt.

HRMS-ESI$^+$ (m/z): $[M+6H]^{6+}$ calcd.: 1310.3466; found: 1310.3422.

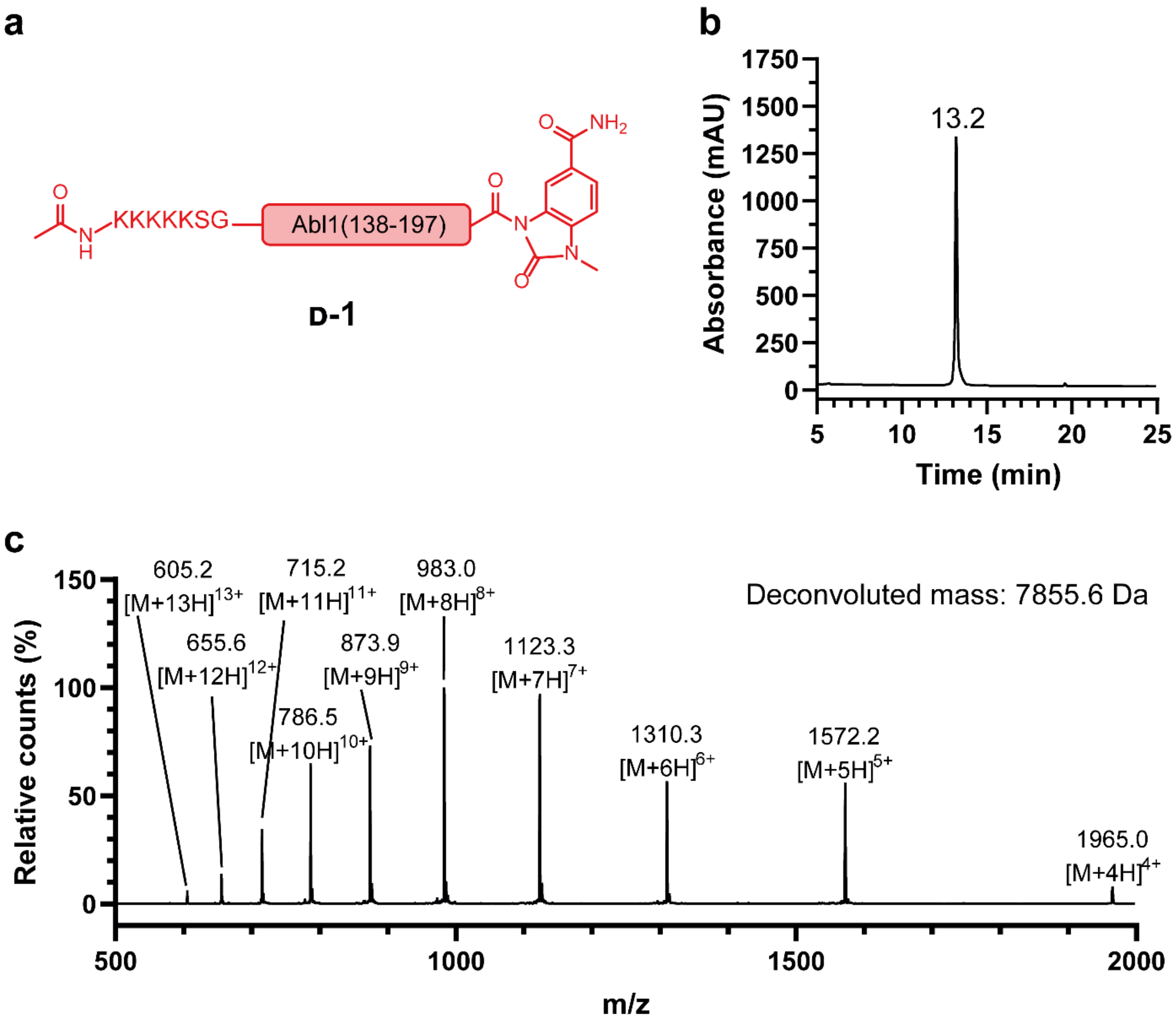

Fig. S3: Characterization of D-1. (**a**) Structure of peptide **D-1**. (**b**) Analytical RP-HPLC chromatogram of purified peptide (HPLC method A, Tab. S1). (**c**) MS-ESI$^+$ spectrum of **D-1** (HPLC Method A, Tab. S1), mass spectra were manually deconvoluted with ESIprot Online.[2]

Synthesis of C-terminal Bcr-Abl SH2 peptide in D-configuration (D-2)

After synthesis and cleavage of peptide **D-2** from the resin, the precipitated peptide was dissolved in H_2O/MeCN (85:15, v/v) with addition of 0.1% TFA and purified via semipreparative RP-HPLC using a linear gradient of 5-40% MeCN with 0.1% TFA over 30 min. After lyophilization, 21.1 mg (3.50 µmol, 14%, 25 µmol scale) of the desired peptide were obtained as TFA salt.

HRMS-ESI$^+$ (m/z): $[M+4H]^{4+}$ calcd.: 1224.1336; found: 1224.1276.

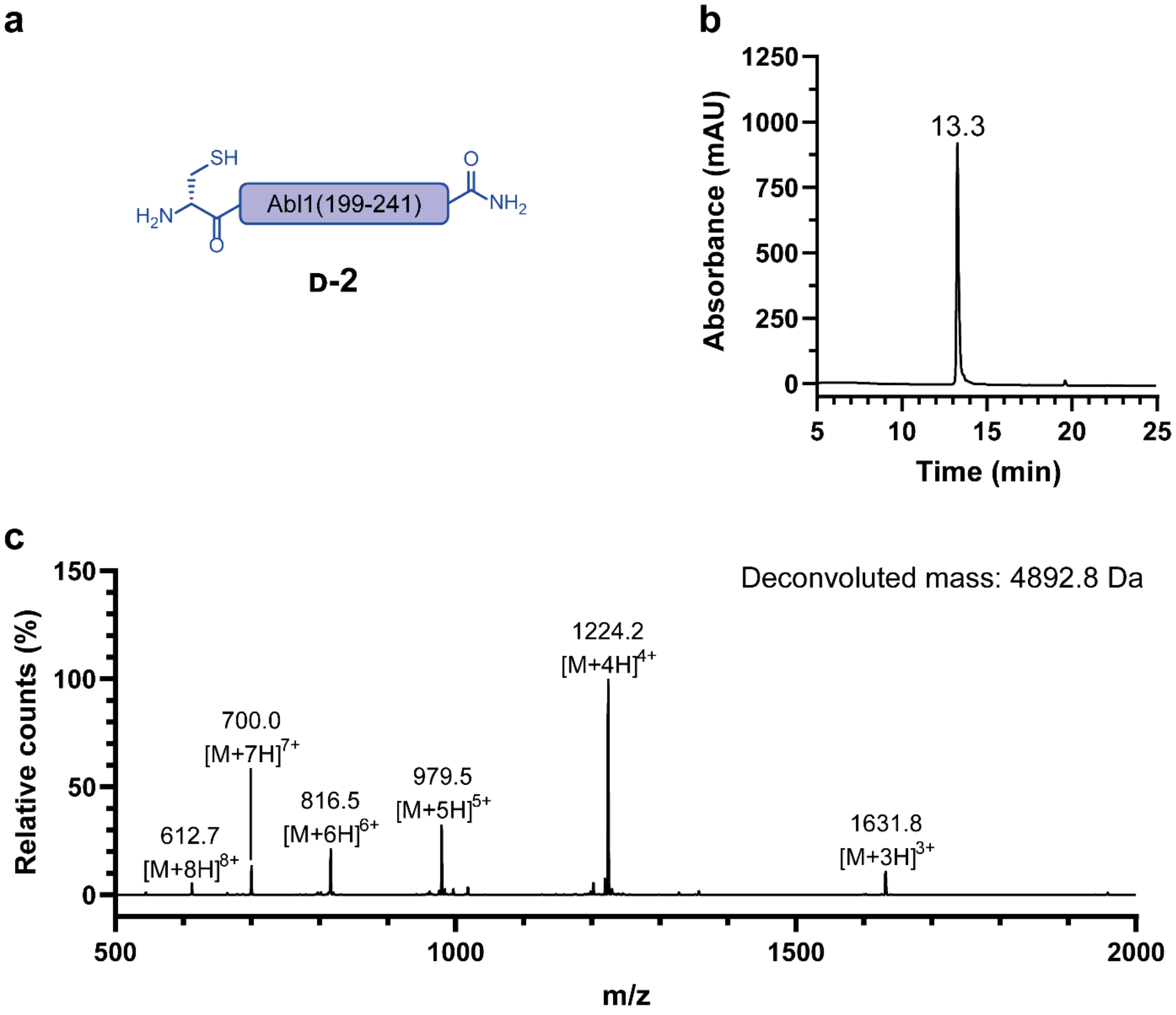

Fig. S4: Characterization of D-2. (**a**) Structure of peptide **D-2**. (**b**) Analytical RP-HPLC chromatogram of purified peptide (HPLC method A, Tab. S1). (**c**) MS-ESI$^+$ spectrum of **D-2** (HPLC Method A, Tab. S1), mass spectra were manually deconvoluted with ESIprot Online.[2]

Synthesis of ligated Bcr-Abl SH2 A198C in D-configuration (D-3)

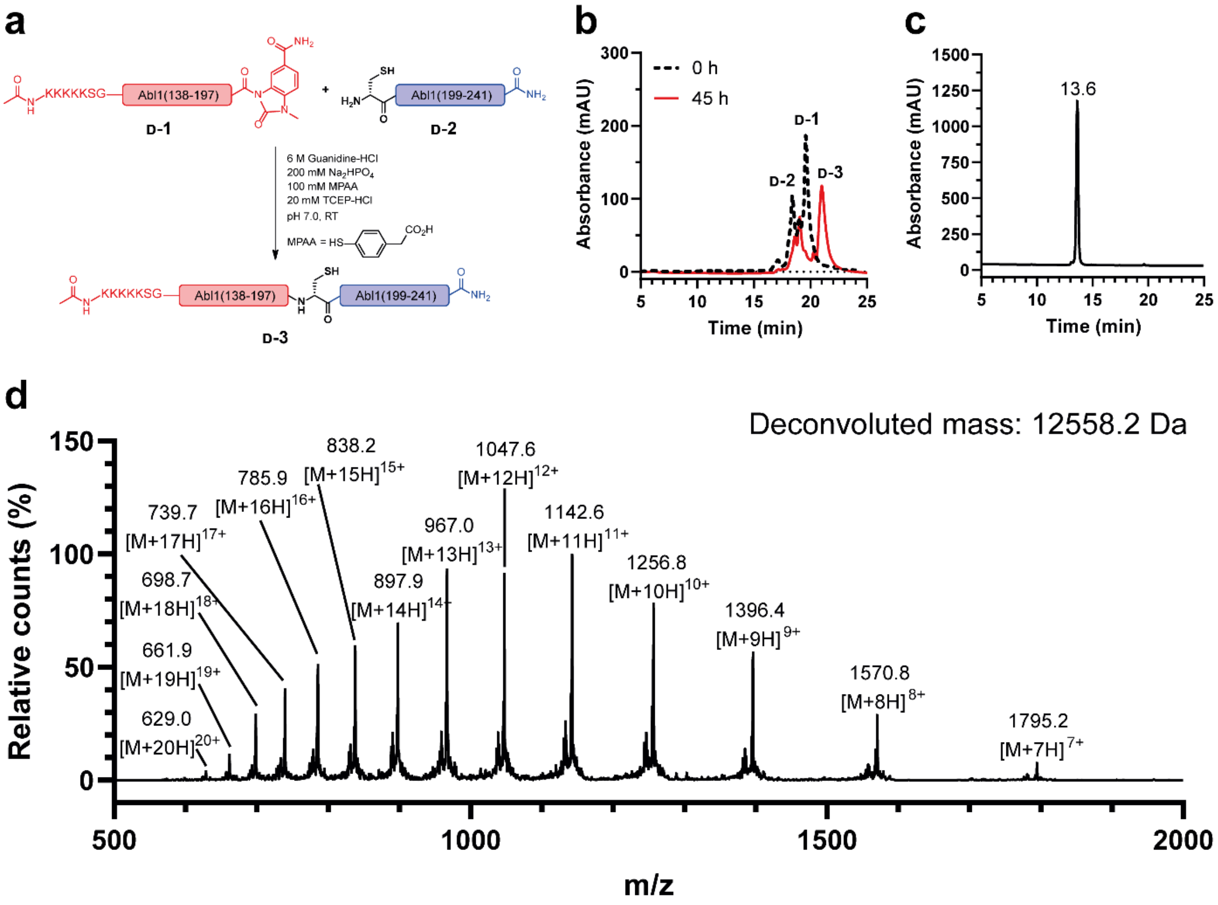

Fig. S5: Characterization of D-3. (**a**) Reaction scheme of the native chemical ligation (NCL) between peptides **D-1** and **D-2** to obtain **D-3**. (**b**) Analytical RP-HPLC chromatogram of NCL crude (HPLC Method B, Tab. S2). (**c**) Analytical RP-HPLC chromatogram of purified ligation product (HPLC Method A, Tab. S1). (**d**) MS-ESI$^+$ spectrum of **D-3** (HPLC Method A, Tab. S1), mass spectra were manually deconvoluted with ESIprot Online.[2]

Peptides **D-1** (1 eq.) and **D-2** (1.1 eq.) were dissolved in 1585 µL of freshly prepared buffer containing 6 M guanidine-HCl, 200 mM Na_2HPO_4, 100 mM MPAA and 20 mM TCEP-HCl. The pH was adjusted to 7.0 using 10 M NaOH and the ligation reaction was carried out at room temperature (RT) for 24 h. Then, a spatula tip of TCEP-HCl powder was added to the mixture. After 30 min, the crude product was dissolved in H_2O/MeCN (70:30, v/v) with addition of 0.1% TFA and purified via semipreparative RP-HPLC using a linear gradient of 5-35% MeCN containing 0.1% TFA over 30 min. After lyophilization, 20.6 mg (1.35 µmol, 42%) of the desired peptide were obtained as TFA salt.

HRMS-ESI$^+$ (m/z): $[M+9H]^{9+}$ calcd.: 1396.3930; found: 1396.3901.

Supplementary Note 6:

ITCs of L-AS25 and L-pY peptide binding to recombinant L-Abl SH2 A198C

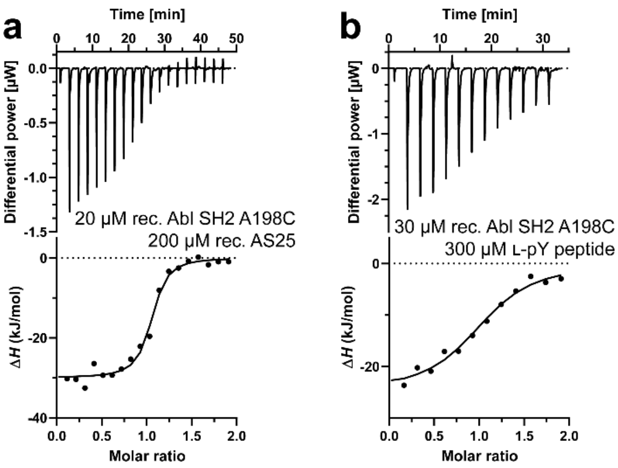

c

	AS25
K_d [nM]	145 ± 53.8
N	1.02 ± 0.017
Δ*H* [kJ/mol]	-29.9 ± 1.22
Δ*G* [kJ/mol]	-39.1
-*T*Δ*S* [kJ/mol]	-9.11

d

	L-pY peptide
K_d [µM]	2.84 ± 1.54
N	1.03 ± 0.083
Δ*H* [kJ/mol]	-24.8 ± 3.78
Δ*G* [kJ/mol]	-31.7
-*T*Δ*S* [kJ/mol]	-6.88

Fig. S6: Binding of recombinantly expressed monobody AS25 and an L-pY peptide to recombinantly expressed Abl SH2 A198C. (**a-b**) Isothermal titration calorimetry (ITC) measurements of (**a**) recombinant L-monobody AS25 and (**b**) L-pY peptide titrated to Abl SH2 A198C. Each panel shows the raw heat signal of an ITC experiment (top) and the integrated calorimetric data of the area of each peak (bottom). The continuous line represents the best fit of the data based on a 1:1 binding model computed from the MicroCal software. Binding parameters including K_d value, stoichiometry (*N*), enthalpy (Δ*H*), free enthalpy (Δ*G*) and -*T*Δ*S* calculated from the fit of each experiment are shown in C-D) below the calorimetric data. A representative measurement of at least two ITC experiments for each monobody is shown.

Supplementary Note 7:

Supplementary figures of the heterochiral Abl SH2-monobody complexes

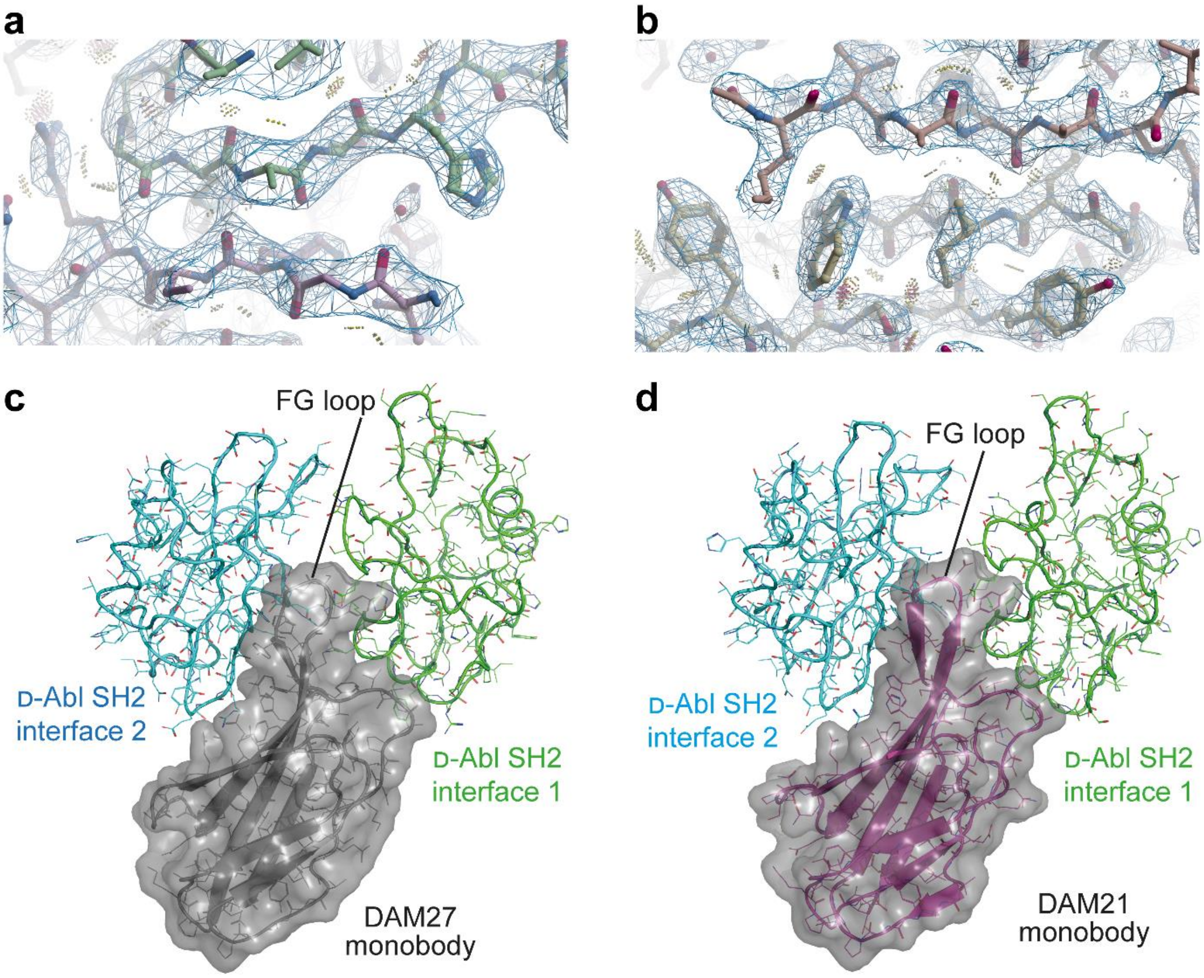

Fig. S7: Structures of the heterochiral D-Abl SH2-monobody complexes. (**a-b**) Image of a portion of the electron density maps (SIGMAA-weighted 2Fo-Fc (2mFo-DFc)) along the D/L-interface of the crystal structures of D-Abl SH2 in complex with (**a**) DAM27 (contour level: 2.0sigma) and (**b**) DAM21 (contour level: 1.5sigma). (**c-d**) Possible interaction surfaces of monobodies (**c**) DAM27 and (**d**) DAM21 binding to synthetic D-Abl SH2.

Supplementary Note 8:

Circular dichroism spectroscopy measurement of rec. L-DAM21 D83A/E85A

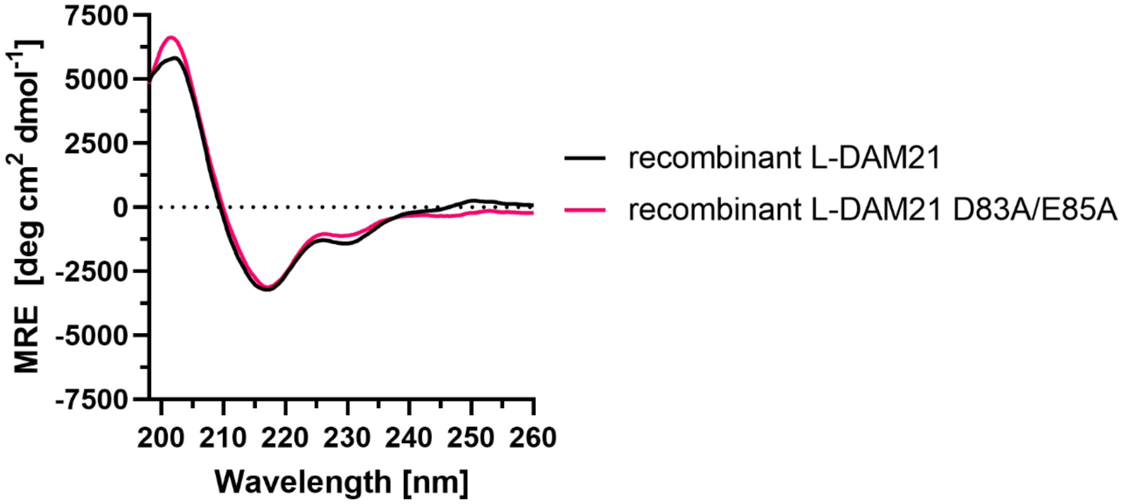

Fig. S8: Averaged far-UV circular dichroism (CD) spectra of recombinantly expressed DAM21 and DAM21 D83A/E85A. Mean residue ellipticity (MRE) was calculated from three independent measurements according to T. E. Creighton.[3]

Tab. S4: Secondary structure content of recombinantly expressed (rec.) DAM21 and DAM21 D83A/E85A. The percentages were calculated with BeStSel based on the obtained CD spectra from three measurements.[4]

**Secondary structure element**	**Rec. DAM21**	**Rec. DAM21 D83A/E85A**
α-helix	0.00 %	0.80 %
β-sheet	49.7 %	44.2 %
β-turn	13.4 %	12.8 %
Others	36.9 %	42.2 %

Supplementary Note 9:

Synthesis of monobodies DAM27 and DAM21

Tab. S5: Sequences of the synthesized monobody peptide fragments.

Peptide	Sequence	Mass [Da]
**4**	Biotin-ASSVPTKLEVVAATPTSLLISWDAPAVTVDHYVITYGETGG-GGGSQEFEVPGSKST-MeNbz-$CONH_2$	6065.7
**5**	H_2N-CTISGLKPGVDYTITVYAYEFYSGEYSHFSPISINYRT-AEGPST-AEGPSTAEGPST-$CONH_2$	5970.5
**6**	Biotin-ASSVPTKLEVVAATPTSLLISWDAPAVTVDHYVITYGETGG-GGGSQEFEVPGSKSTCTISGLKPGVDYTITVYAYEFYSGEYSHFSPI-SINYRT-AEGPSTAEGPSTAEGPST-$CONH_2$	11845.0
**7**	Biotin-ASSVPTKLEVVAATPTSLLISWDAPAVTVDHYVITYGETGG-GGGSQEFEVPGSKSTATISGLKPGVDYTITVYAYEFYSGEYSHFSPI-SINYRT-AEGPSTAEGPSTAEGPST-$CONH_2$	11813.0
**8**	Biotin-ASSVPTKLEVVAATPTSLLISWDAPAVTVDHYVITYGETGG-N-$CONH_2$	4557.2
**9**	H_2N-SPVQEFTVPGSKSTATISGLKPGVDYTITVYASDYYDGEISWY-SPISINYRT-$CONH_2$	5725.3

Synthesis of the N-terminal DAM27 peptide in L-configuration (L-4)

After synthesis of peptide **L-4**, the MeDbz linker activation was performed on resin, which was treated with 4-nitrophenyl chloroformate (2 eq., 20 mM) dissolved in 1000 µL 1,2-dichloroethane (DCE) for 45 min. The reaction was repeated twice. After washing the resin (6 × DCE, 6 × anhydrous DMF), 1000 µL of DIPEA (50 eq., 0.5 M) in anhydrous DMF were added. The reaction proceeded for 15 min, repeated for 30 min and then conducted a third time for 15 min. After cleavage of the peptide from the resin, the precipitated peptide was dissolved in H_2O/MeCN (70:30, v/v) with addition of 0.1% TFA and purified via semipreparative RP-HPLC using a linear gradient of 5-60% MeCN with 0.1% TFA over 30 min. After lyophilization, 8.9 mg (1.39 µmol, 14%, 10 µmol scale) of the desired peptide were obtained as TFA salt.

HRMS-ESI$^+$ (m/z): $[M+5H]^{5+}$ calcd.: 1214.0004; found: 1213.9982.

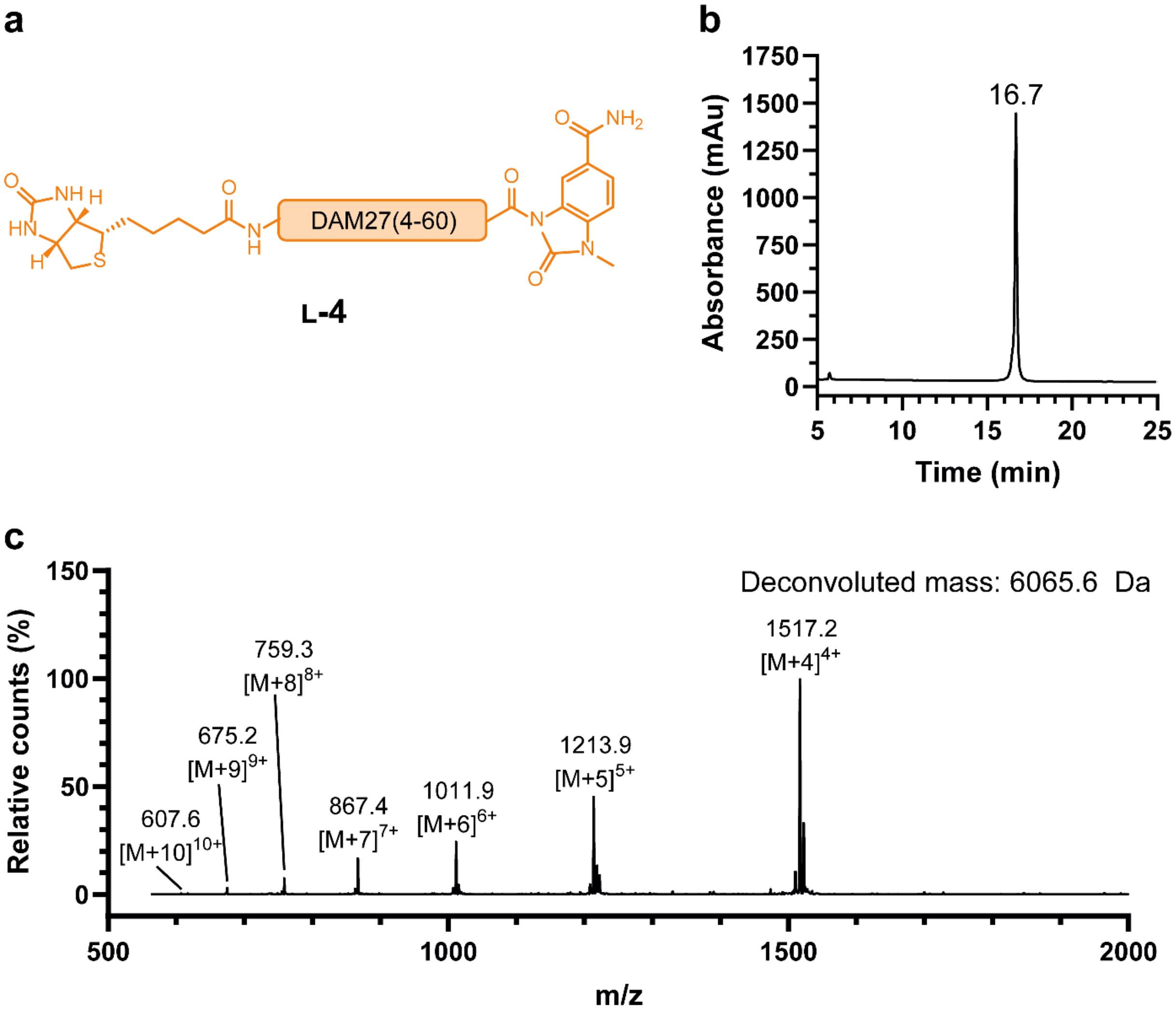

Fig. S9: Characterization of L-4. (**a**) Structure of peptide **L-4**. (**b**) Analytical RP-HPLC chromatogram of purified peptide (HPLC method A, Tab. S1). (**c**) MS-ESI$^+$ spectrum of **L-4** (HPLC Method A, Tab. S1), mass spectra were manually deconvoluted with ESIprot Online.[2]

Synthesis of the N-terminal DAM27 peptide in D-configuration (D-4)

After synthesis of peptide **D-4**, the MeDbz linker activation was performed on resin, which was treated with 4-nitrophenyl chloroformate (2 eq., 20 mM) dissolved in 1500 µL 1,2-dichloroethane (DCE) for 45 min. The reaction was repeated twice. After washing the resin (6 × DCE, 6 × anhydrous DMF), 1500 µL of DIPEA (50 eq., 0.5 M) in anhydrous DMF were added. The reaction proceeded for 15 min, repeated for 30 min and then conducted a third time for 15 min. After cleavage of the peptide from the resin, the precipitated peptide was dissolved in H_2O/MeCN (70:30, v/v) with addition of 0.1% TFA and purified via semipreparative RP-HPLC using a linear gradient of 5-60% MeCN with 0.1% TFA over 30 min. After lyophilization, 9.3 mg (1.45 µmol, 10%, 15 µmol scale) of the desired peptide were obtained as TFA salt.

HRMS-ESI$^+$ (m/z): $[M+4H]^{4+}$ calcd.: 1517.2487; found: 1517.2494.

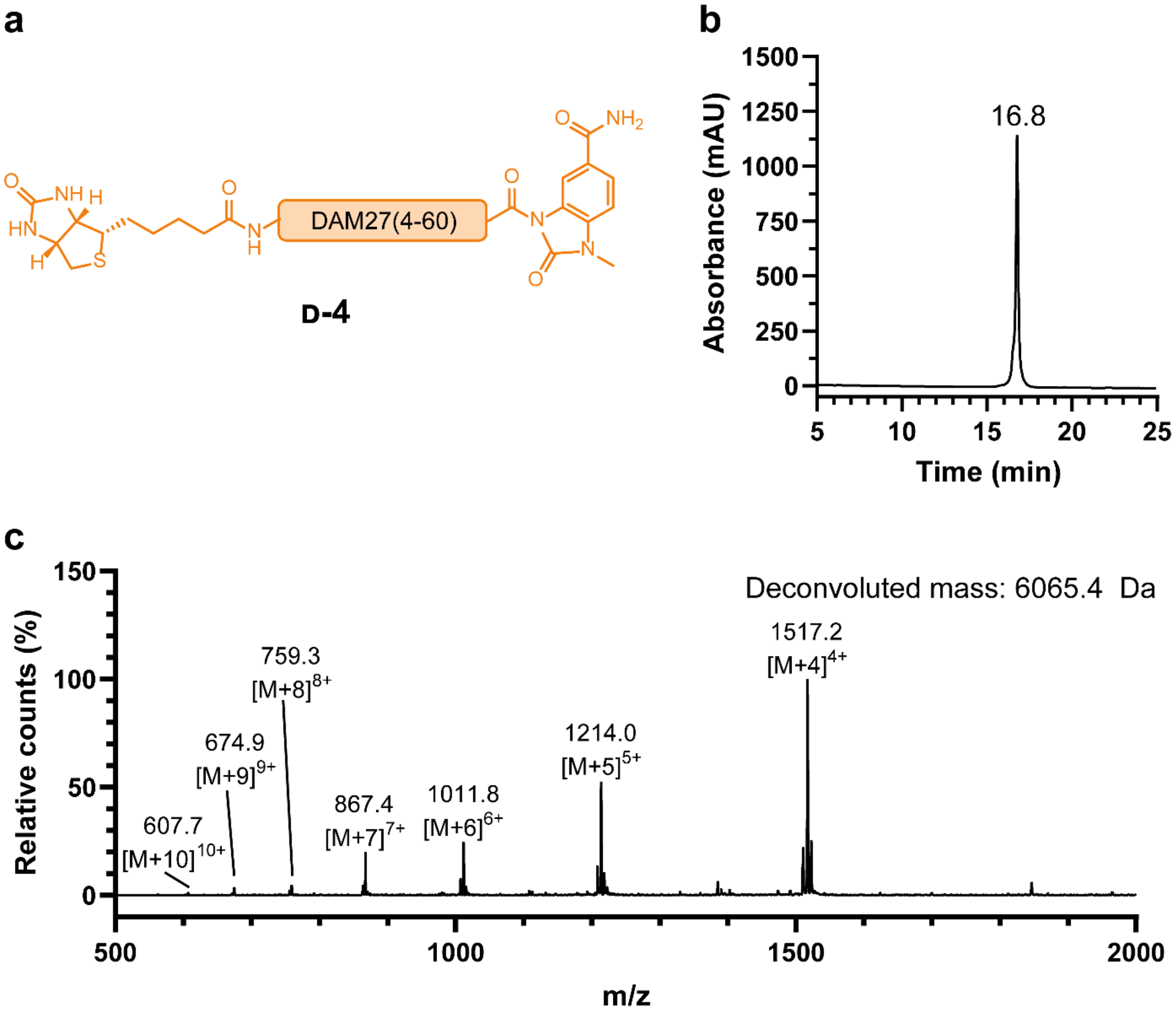

Fig. S10: Characterization of D-4. (**a**) Structure of peptide **D-4**. (**b**) Analytical RP-HPLC chromatogram of purified peptide (HPLC method A, Tab. S1). (**c**) MS-ESI$^+$ spectrum of **D-4** (HPLC Method A, Tab. S1), mass spectra were manually deconvoluted with ESIprot Online.[2]

Synthesis of the C-terminal DAM27 peptide in L-configuration (L-5)

After synthesis and cleavage of peptide **L-5** from the resin, the precipitated peptide was dissolved in H_2O/MeCN (70:30, v/v) with addition of 0.1% TFA and purified via semipreparative RP-HPLC using a linear gradient of 5-60% MeCN with 0.1% TFA over 30 min. After lyophilization, 13.8 mg (2.15 µmol, 11%, 20 µmol scale) of the desired peptide were obtained as TFA salt.

HRMS-ESI$^+$ (m/z): $[M+4H]^{4+}$ calcd.: 1493.4540; found: 1493.4523.

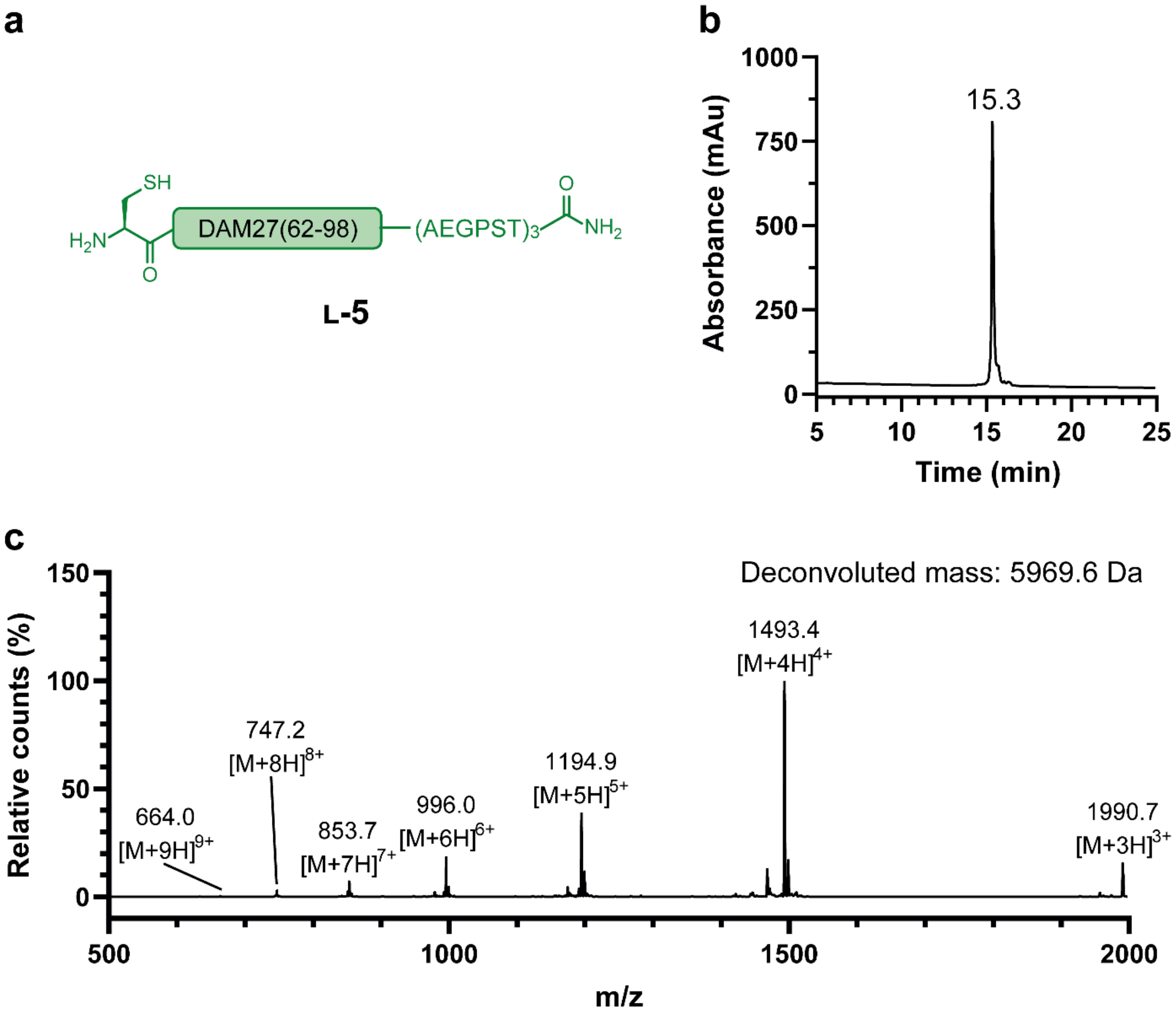

Fig. S11: Characterization of L-5. (**a**) Structure of peptide **L-5**. (**b**) Analytical RP-HPLC chromatogram of purified peptide (HPLC method A, Tab. S1). (**c**) MS-ESI$^+$ spectrum of **L-5** (HPLC Method A, Tab. S1), mass spectra were manually deconvoluted with ESIprot Online.[2]

Synthesis of the C-terminal DAM27 peptide in D-configuration (D-5)

After synthesis and cleavage of peptide **D-5** from the resin, the precipitated peptide was dissolved in H_2O/MeCN (70:30, v/v) with addition of 0.1% TFA and purified via semipreparative RP-HPLC using a linear gradient of 5-60% MeCN with 0.1% TFA over 30 min. After lyophilization, 13.6 mg (2.12 µmol, 7%, 30 µmol scale) of the desired peptide were obtained as TFA salt.

HRMS-ESI$^+$ (m/z): $[M+4H]^{4+}$ calcd.: 1493.4540; found: 1493.4487.

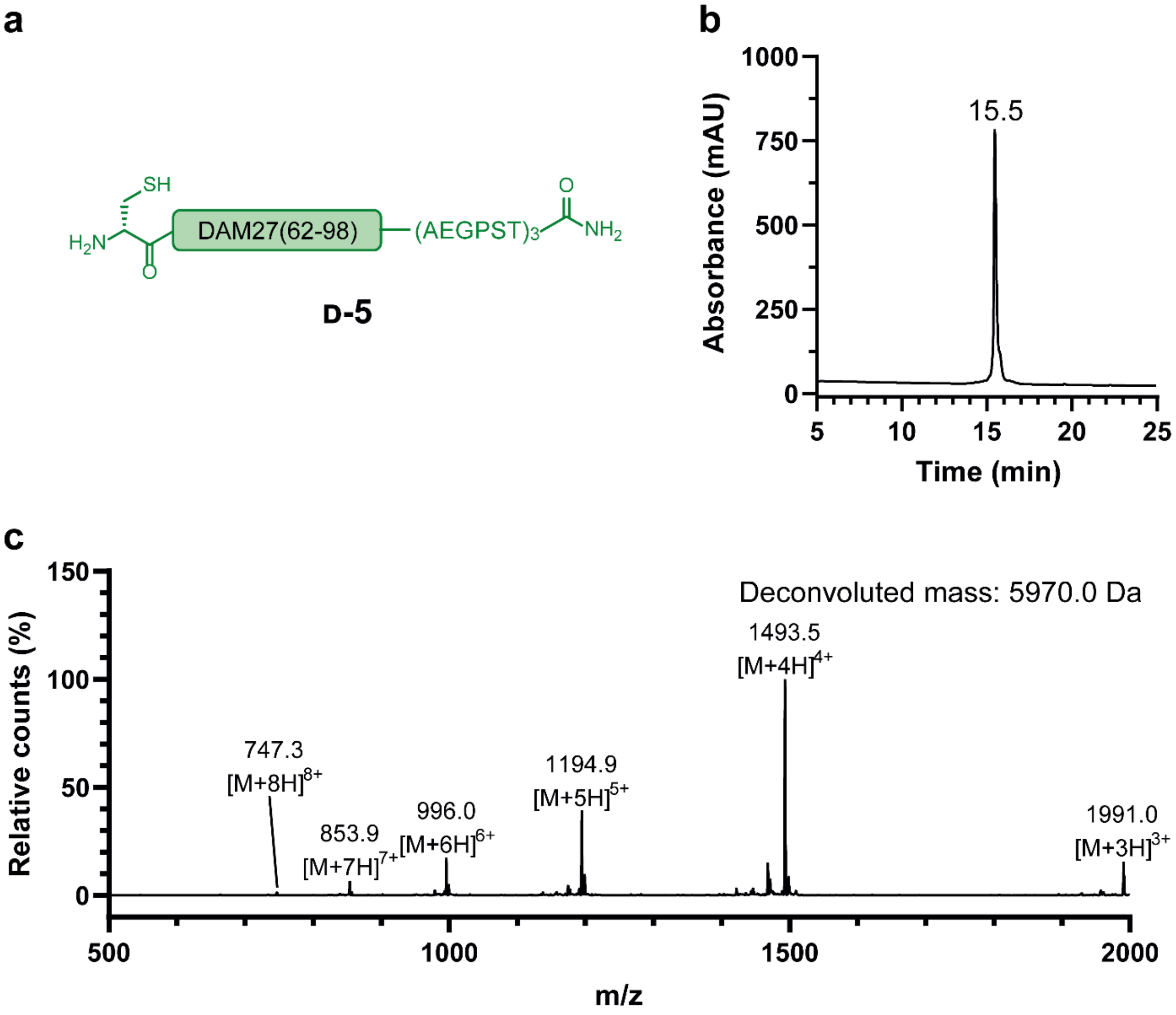

Fig. S12: Characterization of D-5. (**a**) Structure of peptide **D-5**. (**b**) Analytical RP-HPLC chromatogram of purified peptide (HPLC method A, Tab. S1). (**c**) MS-ESI$^+$ spectrum of **D-5** (HPLC Method A, Tab. S1), mass spectra were manually deconvoluted with ESIprot Online.[2]

Synthesis of ligated DAM27 peptide A61C in L-configuration (L-6)

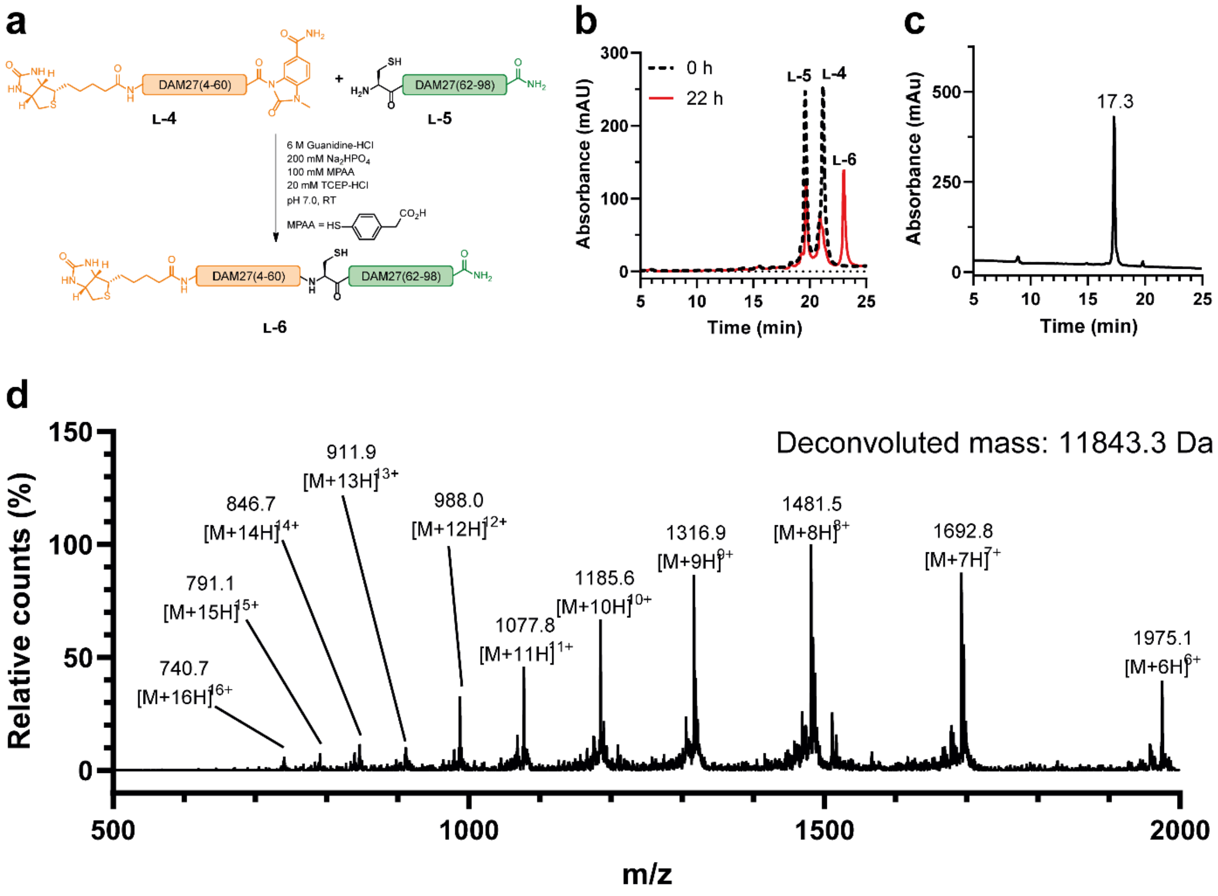

Fig. S13: Characterization of L-6. (**a**) Reaction scheme of the native chemical ligation (NCL) between peptides **L-4** and **L-5** to obtain **L-6**. (**b**) Analytical RP-HPLC chromatogram of NCL crude (HPLC Method C, Tab. S2). (**c**) Analytical RP-HPLC chromatogram of purified ligation product (HPLC Method A, Tab. S1). (**d**) MS-ESI$^+$ spectrum of **L-6** (HPLC Method A, Tab. S1), mass spectra were manually deconvoluted with ESIprot Online.[2]

Peptides **L-4** (8.9 mg, 1.39 µmol, 1 eq.) and **L-5** (10.7 mg, 1.67 µmol, 1.2 eq.) were dissolved in 695 µL of freshly prepared buffer containing 6 M guanidine-HCl, 200 mM Na_2HPO_4, 100 mM MPAA and 20 mM TCEP-HCl. The pH was adjusted to 7.0 using 10 M NaOH and the ligation reaction was carried out at room temperature (RT) for 25 h. Then, a spatula tip of TCEP-HCl powder was added to the mixture. After 30 min, the crude product was dissolved in H_2O/MeCN (50:50, v/v) with addition of 0.1% TFA and purified via semipreparative RP-HPLC using a linear gradient of 5-50% MeCN containing 0.1% TFA over 30 min. After lyophilization, 3.8 mg (0.303 µmol, 22%) of the desired peptide were obtained as TFA salt.

HRMS-ESI$^+$ (m/z): $[M+7H]^{7+}$ calcd.: 1693.1052; found: 1693.1041.

Synthesis of ligated DAM27 peptide A61C in D-configuration (D-6)

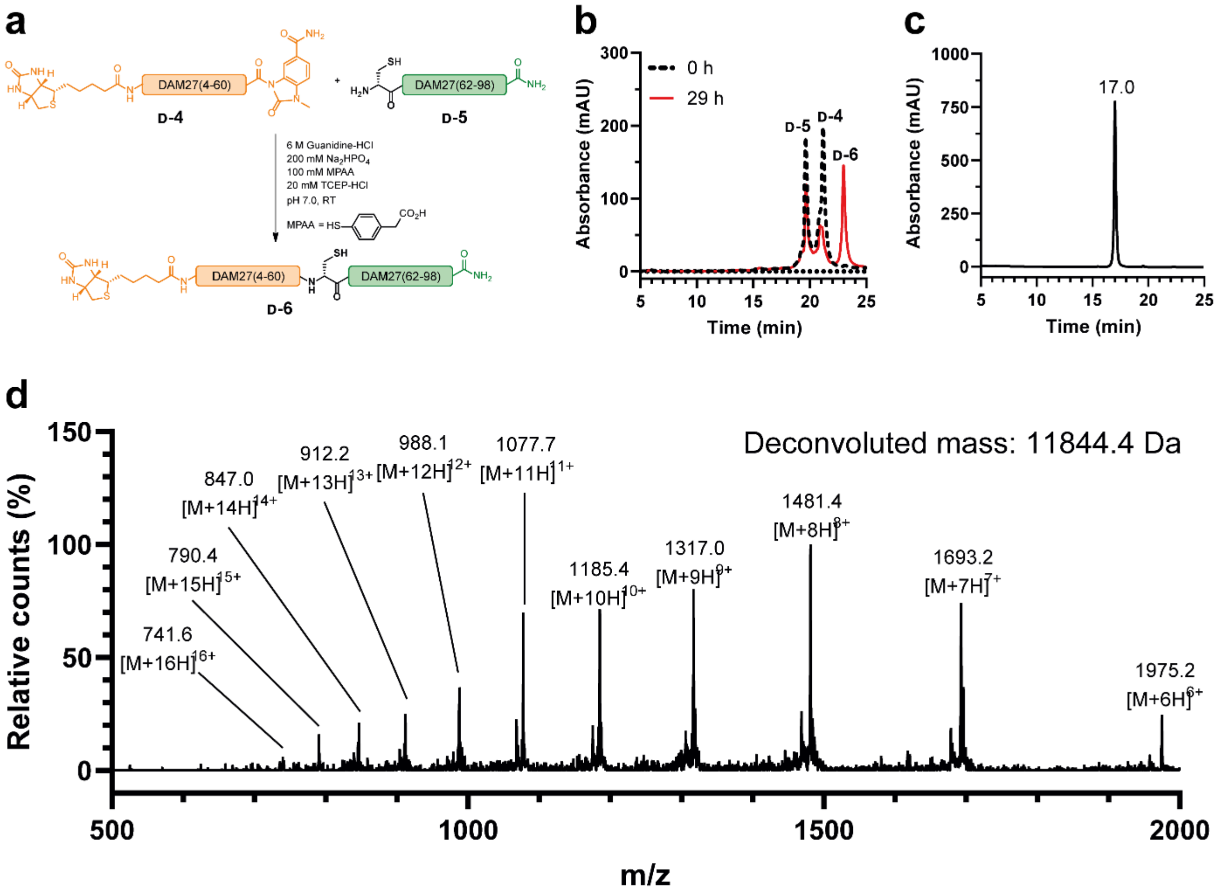

Fig. S14: Characterization of D-6. (**a**) Reaction scheme of the native chemical ligation (NCL) between peptides **D-4** and **D-5** to obtain **D-6**. (**b**) Analytical RP-HPLC chromatogram of NCL crude (HPLC Method C, Tab. S2). (**c**) Analytical RP-HPLC chromatogram of purified ligation product (HPLC Method A, Tab. S1). (**d**) MS-ESI$^+$ spectrum of **D-6** (HPLC Method A, Tab. S1), mass spectra were manually deconvoluted with ESIprot Online.[2]

Peptides **D-4** (9.3 mg, 1.45 µmol, 1 eq.) and **D-5** (11.2 mg, 1.74 µmol, 1.2 eq.) were dissolved in 725 µL of freshly prepared buffer containing 6 M guanidine-HCl, 200 mM Na_2HPO_4, 100 mM MPAA and 20 mM TCEP-HCl. The pH was adjusted to 7.0 using 10 M NaOH and the ligation reaction was carried out at room temperature (RT) for 29 h. Then, a spatula tip of TCEP-HCl powder was added to the mixture. After 30 min, the crude product was dissolved in H_2O/MeCN (50:50, v/v) with addition of 0.1% TFA and purified via semipreparative RP-HPLC using a linear gradient of 5-50% MeCN containing 0.1% TFA over 30 min. After lyophilization, 3.6 mg (0.288 µmol, 20%) of the desired peptide were obtained as TFA salt.

HRMS-ESI$^+$ (m/z): $[M+7H]^{7+}$ calcd.: 1693.1045; found: 1693.0941.

Desulfurization of the DAM27 peptide in L-configuration (L-7)

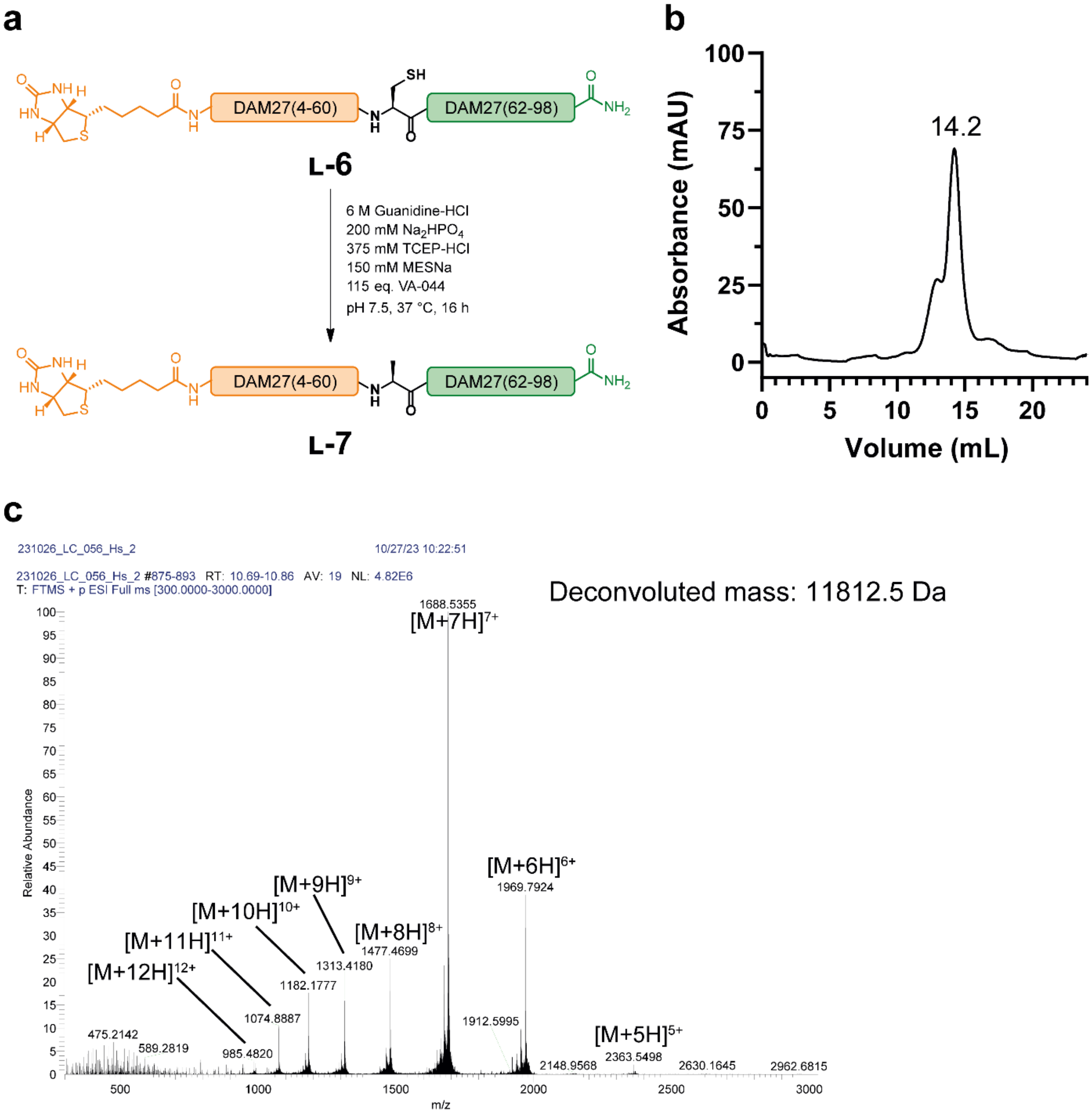

Fig. S15: Characterization of L-7. (**a**) Desulfurization reaction of **L-6** to obtain **L-7**. (**b**) Analytical SEC chromatogram of desulfurized product on a Superdex 75 Increase 10/300 GL column (Cytiva). (**c**) MS-ESI$^+$ spectrum of **L-7**, mass spectra were manually deconvoluted with ESIprot Online.[2]

Peptide **L-6** (1.1 mg, 0.0878 µmol, 325 µM) was dissolved in 270 µL of freshly prepared reaction buffer containing 6 M guanidine-HCl, 200 mM Na_2HPO_4, 375 mM TCEP-HCl, 150 mM MESNa, 115 eq. VA-044 mM. The pH was adjusted to 7.5 using 10 M NaOH and the desulfurization reaction was carried out at 37 °C for 16 h. The desulfurized polypeptide was directly subjected to refolding via dialysis and purified via size exclusion chromatography (SEC) as described in the Methods section of the main manuscript. After purification, the folded monobody (0.428 mg, 0.0362 µmol, 41%) was obtained in solution in 20 mM HEPES, 150 mM NaCl, 0.5 mM TCEP-HCl, pH 7.4 and stored at -80 °C.

HRMS-ESI$^+$ (m/z): $[M+7H]^{7+}$ calcd.: 1688.5378; found: 1688.5355.

Desulfurization of the DAM27 peptide in D-configuration (D-7)

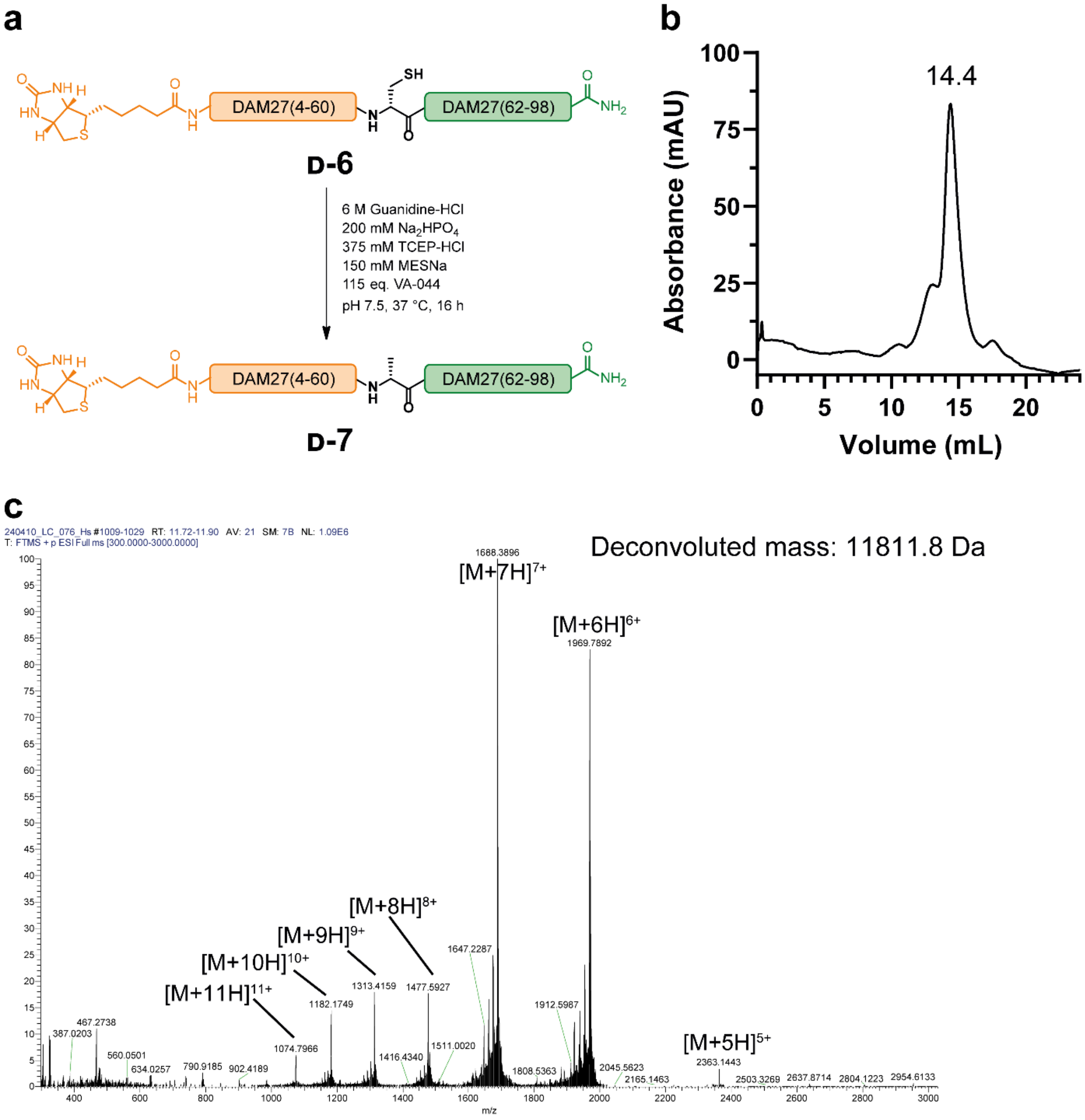

Fig. S16: Characterization of D-7. (**a**) Desulfurization reaction of **D-6** to obtain **D-7**. (**b**) Analytical SEC chromatogram of desulfurized product on a Superdex 75 Increase 10/300 GL column (Cytiva). (**c**) MS-ESI$^+$ spectrum of **D-7**, mass spectra were manually deconvoluted with ESIprot Online.[2]

Peptide **D-6** (1.8 mg, 0.144 µmol, 325 µM) was dissolved in 442 µL of freshly prepared reaction buffer containing 6 M guanidine-HCl, 200 mM Na_2HPO_4, 375 mM TCEP-HCl, 150 mM MESNa, 115 eq. VA-044 mM. The pH was adjusted to 7.5 using 10 M NaOH and the desulfurization reaction was carried out at 37 °C for 16 h. The desulfurized polypeptide was directly subjected to refolding via dialysis and purified via size exclusion chromatography (SEC) as described in the Methods section of the main manuscript. After purification, the folded monobody (1.16 mg, 0.0982 µmol, 68%) was obtained in solution in 20 mM HEPES, 150 mM NaCl, 0.5 mM TCEP-HCl, pH 7.4 and stored at -80 °C.

HRMS-ESI$^+$ (m/z): $[M+7H]^{7+}$ calcd.: 1688.3946; found: 1688.3896.

Synthesis of the N-terminal DAM21 peptide in L-configuration (L-8)

After synthesis and cleavage of peptide **L-8** from the resin, the precipitated peptide was dissolved in H_2O/MeCN (70:30, v/v) with addition of 0.1% TFA and purified via semipreparative RP-HPLC using a linear gradient of 5-60% MeCN with 0.1% TFA over 30 min. After lyophilization, 5.30 mg (1.11 µmol, 11%, 10 µmol scale) of the desired peptide were obtained as TFA salt.

HRMS-ESI$^+$ (m/z): $[M+4H]^{4+}$ calcd.: 1140.0835; found: 1140.0790.

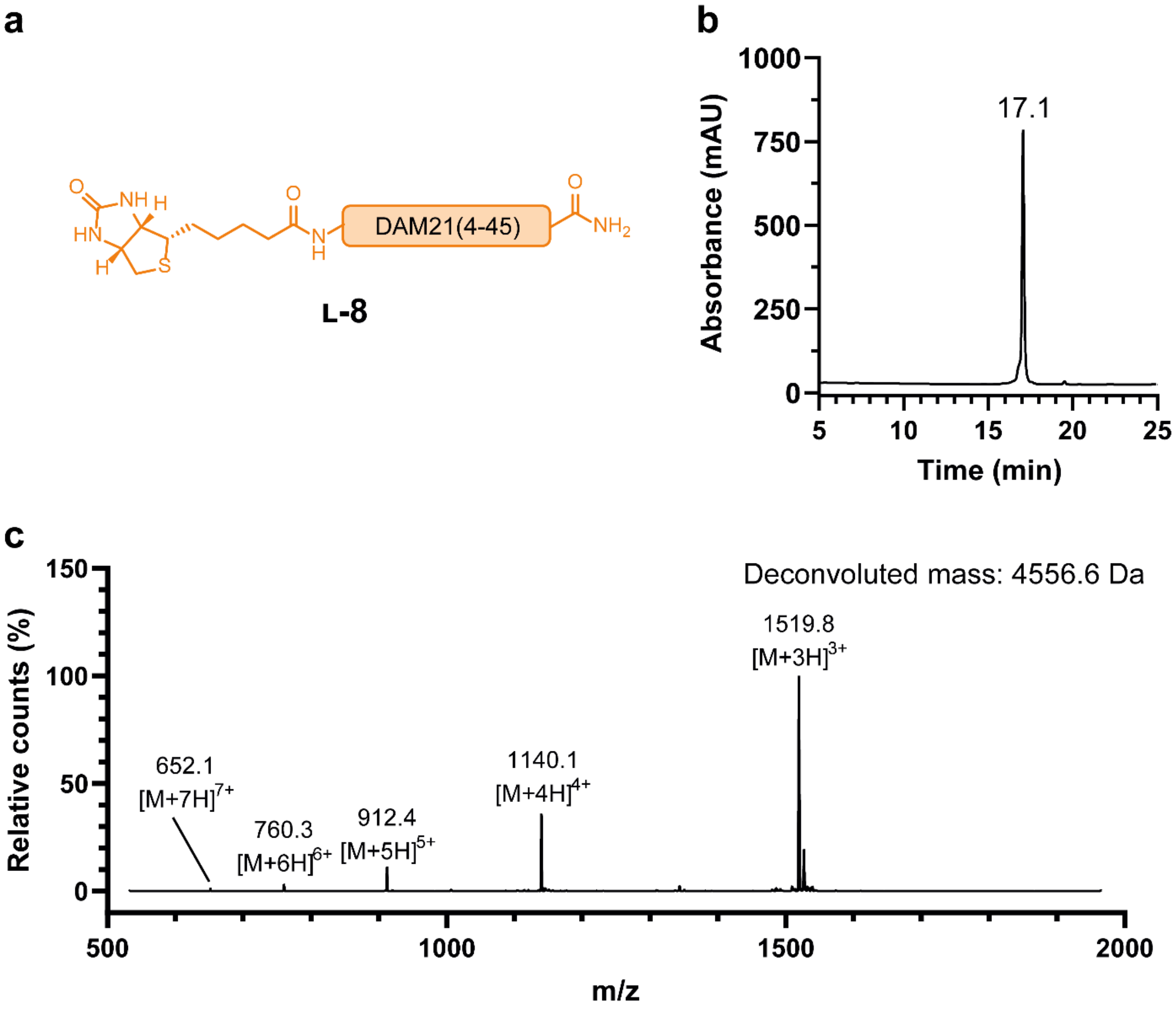

Fig. S17: Characterization of L-8. (**a**) Structure of peptide **L-8**. (**b**) Analytical RP-HPLC chromatogram of purified peptide (HPLC method A, Tab. S1). (**c**) MS-ESI$^+$ spectrum of **L-8** (HPLC Method A, Tab. S1), mass spectra were manually deconvoluted with ESIprot Online.[2]

Synthesis of the N-terminal DAM21 peptide in D-configuration (D-8)

After synthesis and cleavage of peptide **D-8** from the resin, the precipitated peptide was dissolved in H_2O/MeCN (70:30, v/v) with addition of 0.1% TFA and purified via semipreparative RP-HPLC using a linear gradient of 5-60% MeCN with 0.1% TFA over 30 min. After lyophilization, 6.00 mg (1.25 µmol, 13%, 10 µmol scale) of the desired peptide were obtained as TFA salt.

HRMS-ESI$^+$ (m/z): $[M+4H]^{4+}$ calcd.: 1140.0836; found: 1140.0798.

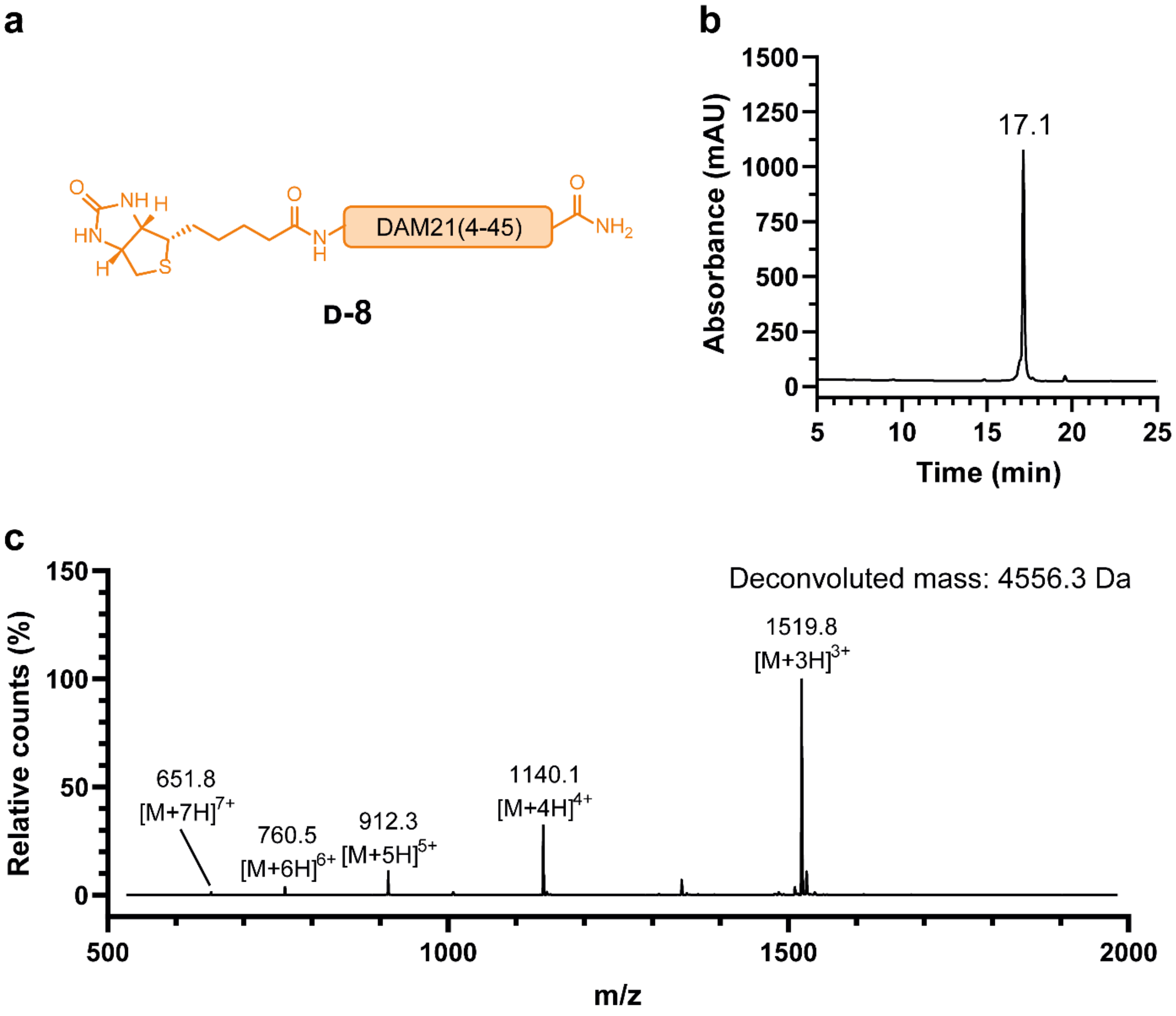

Fig. S18: Characterization of D-8. (**a**) Structure of peptide **D-8**. (**b**) Analytical RP-HPLC chromatogram of purified peptide (HPLC method A, Tab. S1). (**c**) MS-ESI$^+$ spectrum of **D-8** (HPLC Method A, Tab. S1), mass spectra were manually deconvoluted with ESIprot Online.[2]

Synthesis of the C-terminal DAM21 peptide in L-configuration (L-9)

After synthesis, peptide **L-9** was purified using the Peptide Easy Clean (PEC) kit (Belyntic) according to manufacturer's instructions due to solubility and aggregation issues. After lyophilization, 6.40 mg (1.04 µmol, 10%, 10 µmol scale) of the desired peptide were obtained as TFA salt.

HRMS-ESI$^+$ (m/z): $[M+4H]^{4+}$ calcd.: 1432.2105; found: 1432.2096.

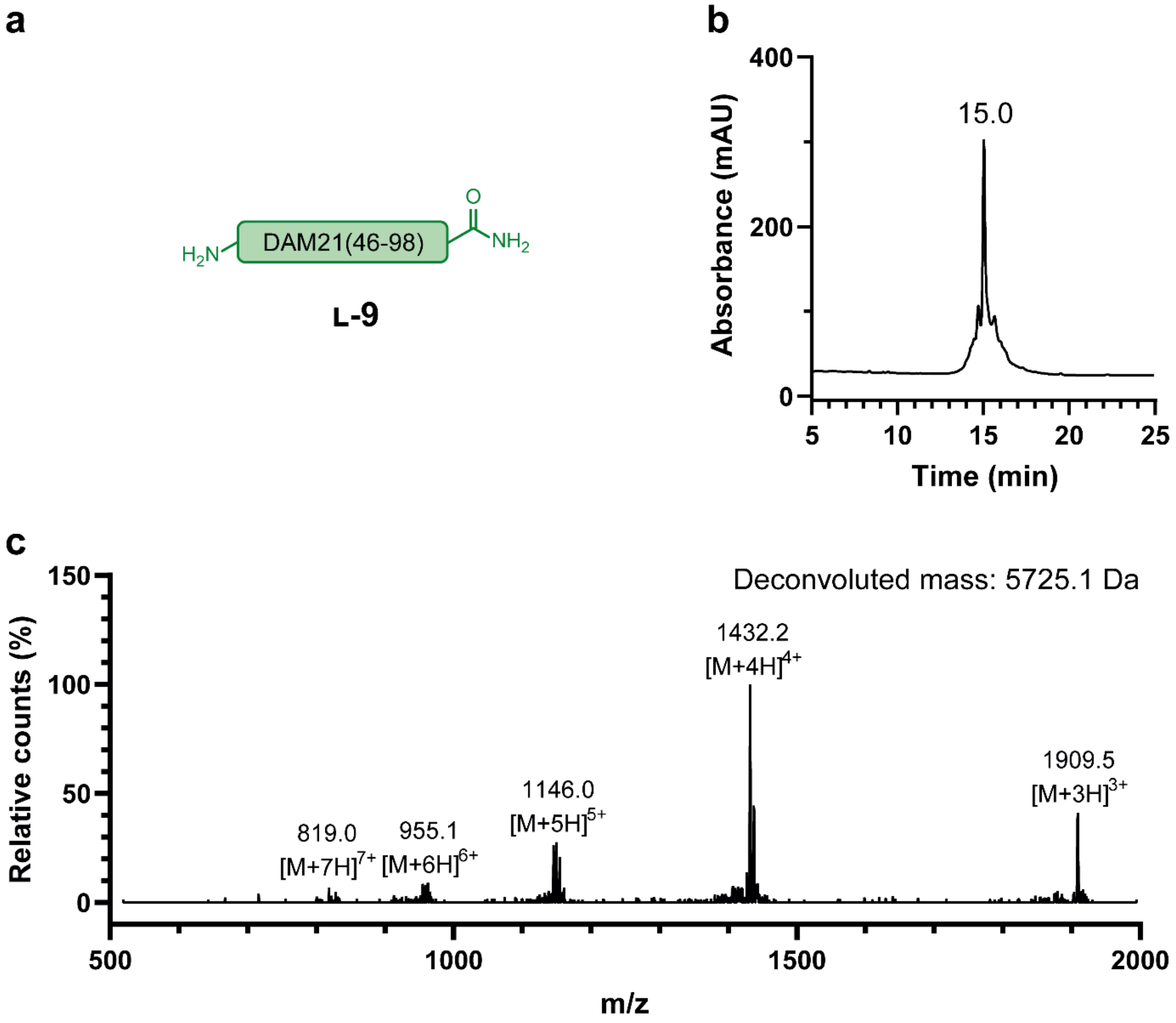

Fig. S19: Characterization of L-9. (**a**) Structure of peptide **L-9**. (**b**) Analytical RP-HPLC chromatogram of purified peptide (HPLC method A, Tab. S1). (**c**) MS-ESI$^+$ spectrum of **L-9** (HPLC Method A, Tab. S1), mass spectra were manually deconvoluted with ESIprot Online.[2]

Synthesis of the C-terminal DAM21 peptide in D-configuration (D-9)

After synthesis, peptide **D-9** was purified using the Peptide Easy Clean (PEC) kit (Belyntic) according to manufacturer's instructions due to solubility and aggregation issues. After lyophilization, 2.40 mg (0.388 µmol, 4%, 10 µmol scale) of the desired peptide were obtained as TFA salt.

HRMS-ESI$^+$ (m/z): $[M+4H]^{4+}$ calcd.: 1432.2105; found: 1432.2053.

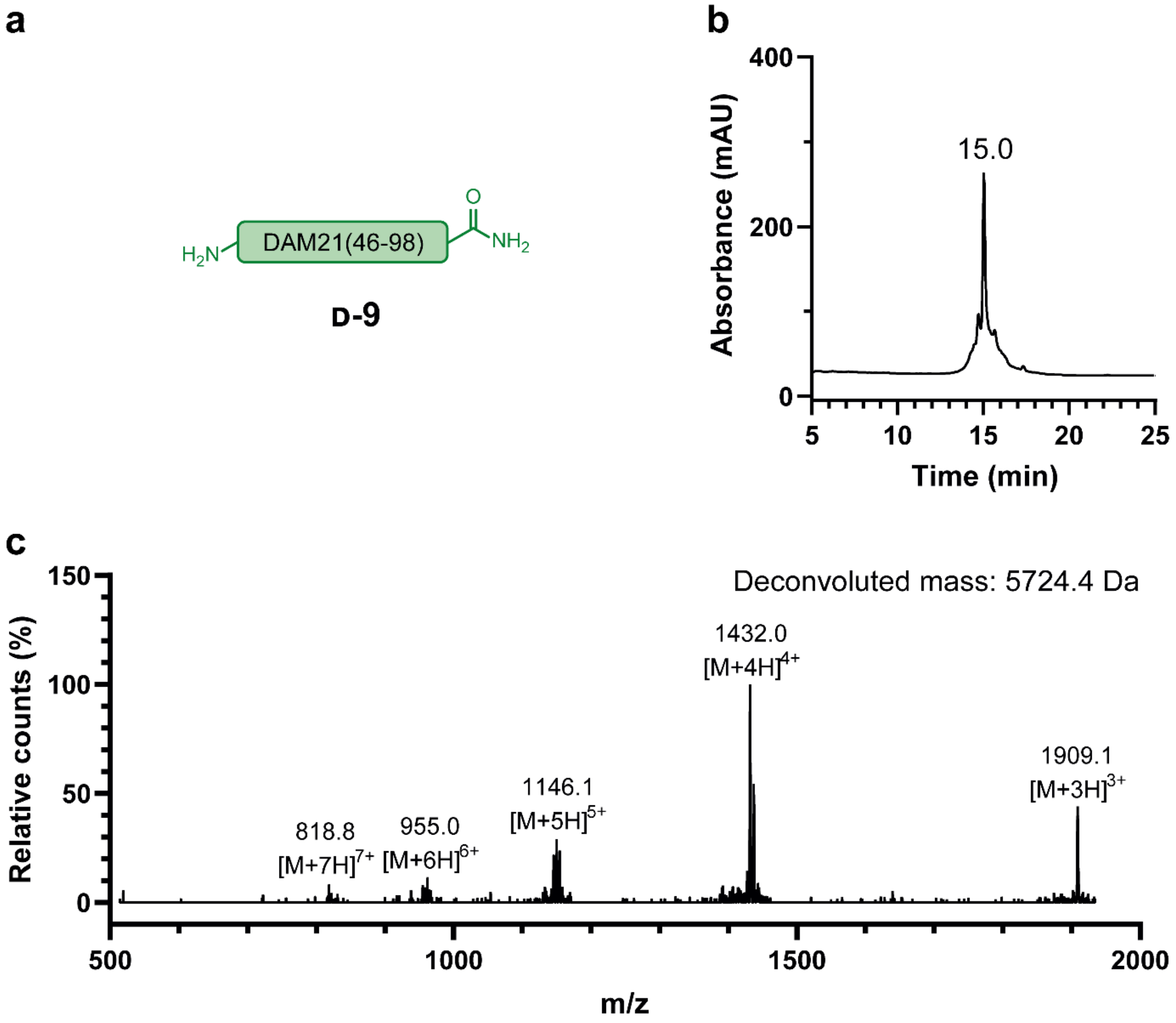

Fig. S20: Characterization of D-9. (**a**) Structure of peptide **D-9**. (**b**) Analytical RP-HPLC chromatogram of purified peptide (HPLC method A, Tab. S1). (**c**) MS-ESI$^+$ spectrum of **D-9** (HPLC Method A, Tab. S1), mass spectra were manually deconvoluted with ESIprot Online.[2]

Supplementary Note 10:

Secondary structure content of synthetic monobodies

Tab. S6: Secondary structure content of recombinantly expressed (rec.) and synthetic (syn.) DAM27 monobodies in L- and D-configuration. The percentages were calculated with BeStSel based on the obtained CD spectra from three measurements.[4]

Secondary structure element	Rec. DAM27	Rec. DAM27-XTEN	Syn. L-DAM27-XTEN	Syn. D-DAM27-XTEN
α-helix	0.00 %	0.00 %	0.00 %	0.00 %
β-sheet	56.3 %	52.4 %	53.6 %	52.9 %
β-turn	11.5 %	13.9 %	12.9 %	13.5 %
Others	32.2 %	33.7 %	33.5 %	33.6 %

Tab. S7: Secondary structure content of recombinantly expressed (rec.) and synthetic (syn.) DAM21 monobodies in L- and D-configuration. The percentages were calculated with BeStSel based on the obtained CD spectra from three measurements.[4]

Secondary structure element	Rec. DAM21	Syn. Split-L-DAM21	Syn. Split-D-DAM21
α-helix	0.00 %	0.00 %	0.00 %
β-sheet	49.7 %	38.5 %	47.6 %
β-turn	13.4 %	16.4 %	14.2 %
Others	36.9 %	45.0 %	38.2 %

Supplementary Note 11:

Unfolding and Refolding of DAM21/27 measured by nanoDSF

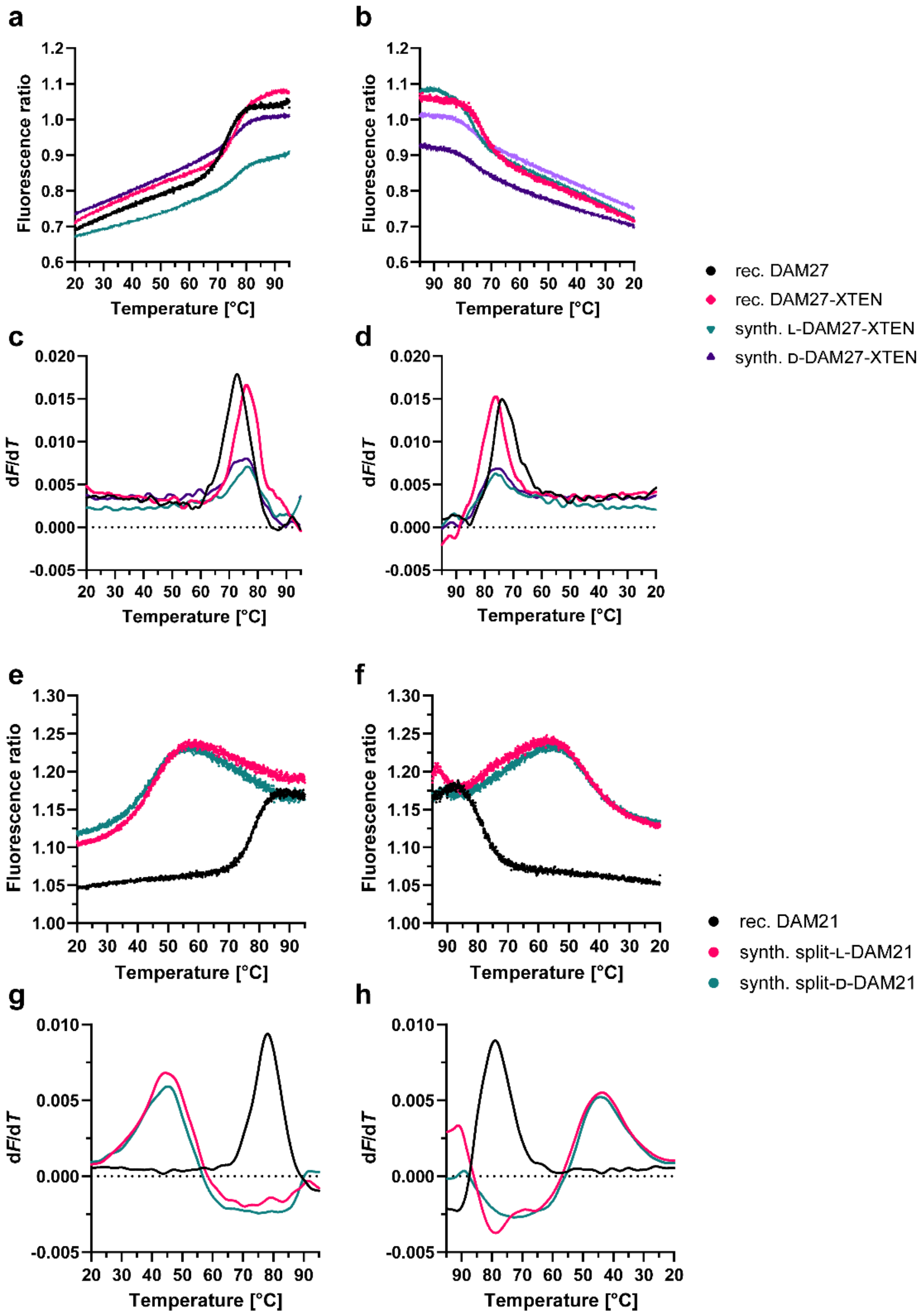

Fig. S21: Unfolding and refolding measurements of recombinantly expressed and refolded, synthetic monobodies DAM27 and DAM21 using nano differential scanning fluorimetry (nanoDSF). (**a-b**) Plot of the fluorescence ratio measured at 330 and 350 nm of DAM27 (**a**) unfolding and (**b**) refolding. (**c-d**) Plot of the first derivative of the measured fluorescence ratio of DAM27 (**c**) unfolding and (**d**) refolding, the maximum indicates the melting temperature (T_m). (**e-f**) Plot of the fluorescence ratio measured at 330 and 350 nm of DAM21 (**e**) unfolding and (**f**) refolding. (**g-h**) Plot of the first derivative of the measured fluorescence ratio of DAM21 (**g**) unfolding and (**h**) refolding, the maximum indicates the melting temperature (T_m).

Supplementary Note 12:

ITC data of rec. L-monobodies DAM21/27 with rec. L-Abl SH2

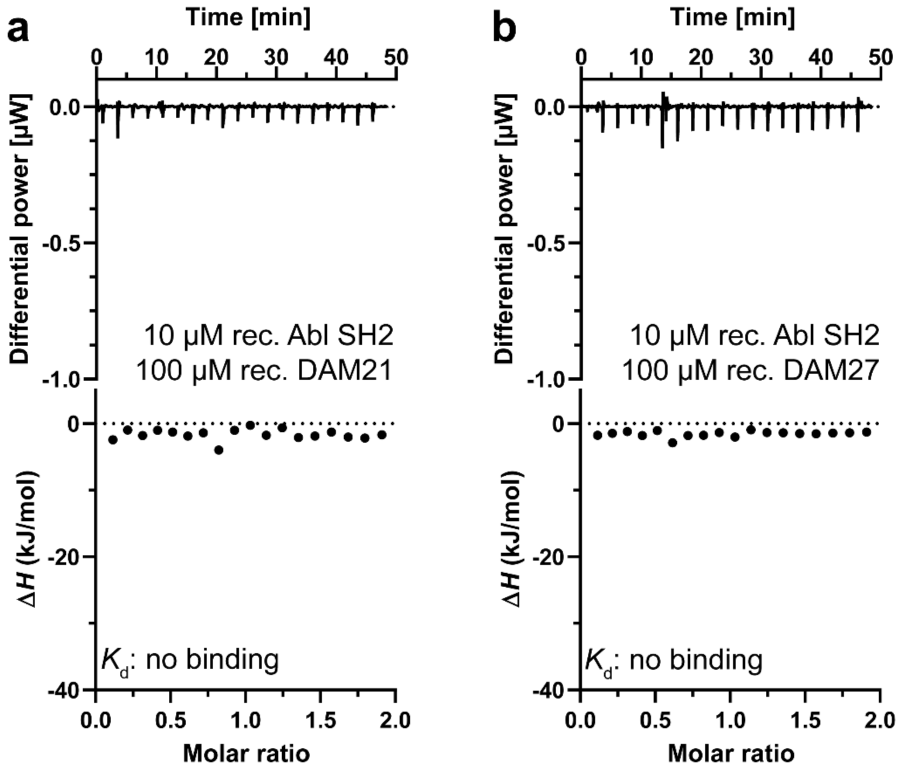

Fig. S22: Binding of recombinantly expressed L-monobodies to recombinantly expressed L-Abl SH2. (**a-b**) Isothermal titration calorimetry (ITC) measurements of (**a**) L-DAM21 and (**b**) L-DAM27 titrated to the L-Abl SH2 domain. Each panel shows the raw heat signal of an ITC experiment (top) and the integrated calorimetric data of the area of each peak (bottom).

Supplementary Note 13:

Sequence alignment of βC/βD strands of SH2 domains

Tab. S8: Multiple sequence alignment of the βC and βD strands of SH2 domains belonging to different SH2 domain-containing tyrosine kinase families. The βC and βD strands are coloured in red and green, respectively, while the CD loop in between is black. The Arg189 of ABL1, which is important for D-monobody binding via ionic interactions, is not conserved and coloured in bold green to highlight the different amino acid sequences in this position.

Protein	Sequence
	βC βD
ABL1	RSISLRY----EG---------**R**VYHYRINT
ABL2	LSISLRY----EG---------**R**VYHYRINT
LCK	FSLSVRDFDQNQG---------**E**VVKHYKIR
YES	YSLSIRDWDE-IRGD-------**N**VKHYKIRK
BTK	YTVSVFAKSTGDPQG-------**V**IRHYVVCS
TEC	YTVSLYTKFGGEGSS-------**G**FRHYHIKE
SHP1	FVLSVLS----DQPKAGPGSPL**R**VTHIKV-M
SHP2	FVLSVRT----GDDKGESNDGK**S**KVTHVMIR
CSK	YTLCVSC----DG---------**K**VEHYRIMY
CRKL	YVLSVSE----NS---------**R**VSHYIINS
SHB	YSLSLRS----NQ---------**G**FMHMKLAK
SHC4	YVLSGLQ----GG---------**Q**AKHLLL-V
VAV1	FAISIKY----NV---------**E**VKHIKIMT
SHIP2	FALCVLY----QK---------**H**VHTYRI--
GRB2	FSLSVKF----GN---------**D**VQHFKVLR
SHC1	YVLTGLQ----SG---------**Q**PKHLLL--
SH2B1	YVLTFNF----QG---------**K**AKHLRLSL
SH2B2	YVLTFNF----QG---------**K**AKHLRLSL

Supplementary Note 14:

Pulldown of BCR::ABL1 from K562 cells with D-DAM21/27

Tab. S9: Proteins identified by mass spectrometry and statistical analysis via MaxQuant 2.5.1.0 and Autonomics (R package version 1.13.21)[5] after pulldown of BCR::ABL1 from K562 cell lysates using L-/D-DAM21. A $\log_2$(ratio) above 1.00 of D- vs. L-monobody DAM21 correlates to a ratio above 2.00 and a higher abundance of the protein when the pulldown was performed with the D-monobody compared to its L-counterpart. Calculated *p*-values from three independent experiments per monobody were FDR-corrected (Benjamini-Hochberg procedure) and a *p*-value below 0.05, which corresponds to $-\log10(p)$ above 1.3, was considered statistically significant. BCR::ABL1 interactors were determined through the BioGRID database[6] and matched with the list of identified proteins in this pulldown experiment. The colour coding represents the protein groups in the volcano plot (Fig. 9H).

DAM21	Protein	$\log_2$(ratio)	*p*-value	$-\log_{10}(p)$
BCR::ABL1				
	ABL1	1.75	0.00079	3.10
	BCR	3.99	0.00001	4.96
BCR::ABL1 interactors				
	AP2A1	1.92	0.04915	1.31
	EMD	1.19	0.00040	3.39
	EPS15	2.38	0.00005	4.35
	FUS	2.84	0.00008	4.08
	HSPD1	1.17	0.00126	2.90
	PRKDC	0.96	0.00049	3.31
	PSMA4	3.99	3×10^{-8}	7.48
	SPTA1	2.00	0.01281	1.89
	XPO1	1.44	0.00228	2.64
	YTHDC1	1.86	0.00387	2.41
BCR::ABL1 interactors with SH2 domains				
	STAT1	1.24	0.01287	1.89
Proteins containing SH2 domains				
	BTK	-0.15	0.72579	0.14
	SH2B1	-0.28	0.68637	0.16
	TYK2	-0.57	0.11873	0.93

Tab. S10: Proteins identified by mass spectrometry and statistical analysis via MaxQuant 2.5.1.0 and Autonomics (R package version 1.13.21)[5] after pulldown of BCR::ABL1 from K562 cell lysates using L-/D-DAM27. A $\log_2$(ratio) above 1.00 of D- vs. L-monobody DAM27 correlates to a ratio above 2.00 and a higher abundance of the protein when the pulldown was performed with the D-monobody compared to its L-counterpart. Calculated *p*-values from three independent experiments per monobody were FDR-corrected (Benjamini-Hochberg procedure) and a *p*-value below 0.05, which corresponds to -log10(*p*) above 1.3, was considered statistically significant. BCR::ABL1 interactors were determined through the BioGRID database[6] and matched with the list of identified proteins in this pulldown experiment. The colour coding represents the protein groups in the volcano plot (Fig. 9I).

DAM27	Protein	$\log_2$(ratio)	*p*-value	$-\log_{10}(p)$
BCR::ABL1				
	ABL1	0.52	0.41340	0.38
	BCR	1.83	0.00638	2.20
BCR::ABL1 interactors				
	CDK1	1.08	0.06909	1.16
	CUL4B	1.64	0.00458	2.34
	EMD	1.16	0.00069	3.16
	EPS15	1.56	0.01024	1.99
	GTF3C4	2.40	0.00032	3.50
	HSPD1	1.19	0.00150	2.83
	MTOR	2.39	7×10^{-6}	5.18
	SPTA1	2.11	0.01327	1.88
	TRIM25	1.15	0.02354	1.63
	XPO1	0.89	0.06834	1.17
BCR::ABL1 interactors with SH2 domains				
	STAT1	2.03	0.00088	3.05
Proteins containing SH2 domains				
	BTK	0.07	0.92595	0.03
	CSK	-0.32	0.55892	0.25
	SH2B1	0.16	0.88491	0.05

Supplementary Note 15:

Binding of monobodies to BCR::ABL1 in permeabilized K562 cells

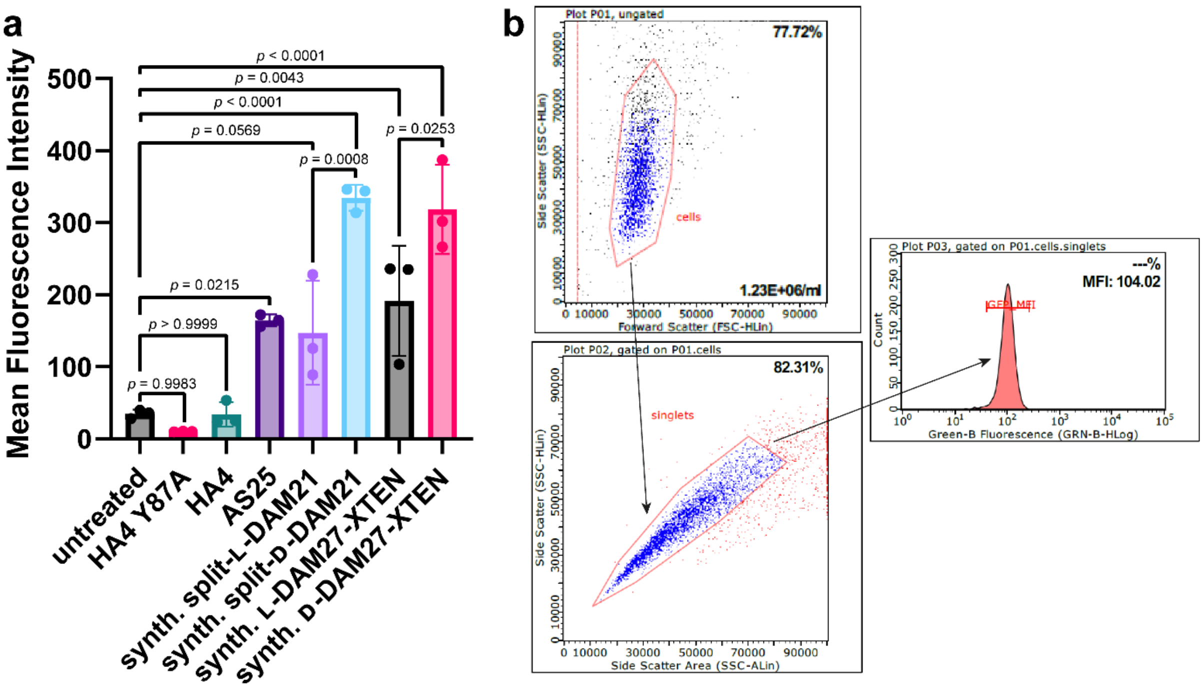

Fig. S23: FACS-based binding assay of monobodies in permeabilized K562 cells. (**a**) Mean fluorescence intensity (MFI) plot of biotinylated monobodies (2 µM) binding to BCR::ABL1 in permeabilized K562 cells measured via flow cytometry and detection of Biotin through AlexaFluor488-coupled streptavidin. Three independent experiments were performed (depicted as dots) and averaged. Error bars represent the standard deviation (SD) and statistical analysis was done with a one-way ANOVA and Sidak's test. The calculated p values are depicted in (**a**) and were considered statistically significant below a value of 0.05. F values and degrees of freedom were 24.38 and 23. Source data of this figure are provided as a Source Data file. (**b**) Gating strategy to measure MFI of permeabilized K562 samples after monobody treatment shown in (**a**).

Supplementary References

1 Gietz, R. D. & Woods, R. A. Genetic transformation of yeast. *Biotechniques* **30**, 816-820, 822-816, 828 passim, doi:10.2144/01304rv02 (2001).

2 Winkler, R. ESIprot: a universal tool for charge state determination and molecular weight calculation of proteins from electrospray ionization mass spectrometry data. *Rapid Commun. Mass Spectrom.* **24**, 285-294, doi:10.1002/rcm.4384 (2010).

3 Creighton, T. E. *Protein structure : a practical approach*. Repr. with corrections edn, (IRL, 1990).

4 Micsonai, A. *et al.* BeStSel: a web server for accurate protein secondary structure prediction and fold recognition from the circular dichroism spectra. *Nucleic Acids Res* **46**, W315-W322, doi:10.1093/nar/gky497 (2018).

5 Bhagwat, A., Cotton, R., Hayat, S., Graumann, J. (2024). autonomics: Unified statistal Modeling of Omics Data, R package version 1.13.21, commit e7d3fa4db08f112530160d3b8e5a29e609bacb4f, (https://https://gitlab.uni-marburg.de/fb20/ag-graumann/software/autonomics).

6 Oughtred, R. *et al.* The BioGRID database: A comprehensive biomedical resource of curated protein, genetic, and chemical interactions. *Protein Sci* **30**, 187-200, doi:10.1002/pro.3978 (2021).
