# Supplementary material for: Development of mirror-image monobodies targeting the oncogenic BCR::ABL1 kinase
Source: Nat Commun. 2024 Dec 23;15:10724. doi: 10.1038/s41467-024-54901-y (PMC11666773; doi:10.1038/s41467-024-54901-y)
Supplement: Supplementary file 2 — Reporting Summary [file 41467_2024_54901_MOESM2_ESM.pdf]

nature portfolio

Corresponding author(s): Olalla Vázquez, Oliver Hantschel

Last updated by author(s): Oct 16, 2024

Reporting Summary

Nature Portfolio wishes to improve the reproducibility of the work that we publish. This form provides structure for consistency and transparency in reporting. For further information on Nature Portfolio policies, see our Editorial Policies and the Editorial Policy Checklist.

Statistics

For all statistical analyses, confirm that the following items are present in the figure legend, table legend, main text, or Methods section.

n/a	Confirmed	
☐	☒	The exact sample size (*n*) for each experimental group/condition, given as a discrete number and unit of measurement
☐	☒	A statement on whether measurements were taken from distinct samples or whether the same sample was measured repeatedly
☐	☒	The statistical test(s) used AND whether they are one- or two-sided<br>*Only common tests should be described solely by name; describe more complex techniques in the Methods section.*
☒	☐	A description of all covariates tested
☒	☐	A description of any assumptions or corrections, such as tests of normality and adjustment for multiple comparisons
☐	☒	A full description of the statistical parameters including central tendency (e.g. means) or other basic estimates (e.g. regression coefficient) AND variation (e.g. standard deviation) or associated estimates of uncertainty (e.g. confidence intervals)
☐	☒	For null hypothesis testing, the test statistic (e.g. *F*, *t*, *r*) with confidence intervals, effect sizes, degrees of freedom and *P* value noted<br>*Give P values as exact values whenever suitable.*
☒	☐	For Bayesian analysis, information on the choice of priors and Markov chain Monte Carlo settings
☒	☐	For hierarchical and complex designs, identification of the appropriate level for tests and full reporting of outcomes
☒	☐	Estimates of effect sizes (e.g. Cohen's *d*, Pearson's *r*), indicating how they were calculated

Our web collection on statistics for biologists contains articles on many of the points above.

Software and code

Policy information about availability of computer code

Data collection	Crystallographic diffraction data were processed and scaled with the XDS package (Kabsch, 1993). Proteomics data was measured by the "DDA PASEF standard_1.1sec_cycletime" method (Bruker Daltonics).
Data analysis	Flow cytometry data was analysed using guavaSoft v4.0 (Luminex), ITC data with MicroCal PEAQ-ITC Analysis Software v1.40 (Malvern Panalytical), SDS-PAGE gels and Western blots with Image Studio v5.2.5 (LI-COR).<br>Crystallography software: Molecular replacement, manual model building, B-factor refinement, solvent addition, energy-minimization: Phaser and Coot (Phenix version 1.17.1); Refinement of structures using phenix.refine; Molecular graphics: PyMOL 2.5.4 (DeLano Scientific).<br>Proteomics data analysis was performed using MaxQuant 2.5.1.0 (MPI of Biochemistry, Germany) and statistical analysis of proteomics data was done with Autonomics (R package version 1.13.21).<br>Data was plotted and statistical analysis was done with Prism v8.4.3 (GraphPad).

For manuscripts utilizing custom algorithms or software that are central to the research but not yet described in published literature, software must be made available to editors and reviewers. We strongly encourage code deposition in a community repository (e.g. GitHub). See the Nature Portfolio guidelines for submitting code & software for further information.

Data

Policy information about availability of data

All manuscripts must include a data availability statement. This statement should provide the following information, where applicable:

- Accession codes, unique identifiers, or web links for publicly available datasets
- A description of any restrictions on data availability
- For clinical datasets or third party data, please ensure that the statement adheres to our policy

The crystal structures of the DAM27:D-Abl SH2 and DAM21:D-Abl SH2 complexes were deposited at Protein Data Bank (entries 9F00 and 9F01). The mass spectrometry proteomics data have been deposited to the ProteomeXchange Consortium via the PRIDE partner repository with the dataset identifier PXD056009. Supplementary Information and Source Data are provided with this paper.

Research involving human participants, their data, or biological material

Policy information about studies with human participants or human data. See also policy information about sex, gender (identity/presentation), and sexual orientation and race, ethnicity and racism.

Reporting on sex and gender	n/a
Reporting on race, ethnicity, or other socially relevant groupings	n/a
Population characteristics	n/a
Recruitment	n/a
Ethics oversight	n/a

Note that full information on the approval of the study protocol must also be provided in the manuscript.

Field-specific reporting

Please select the one below that is the best fit for your research. If you are not sure, read the appropriate sections before making your selection.

☒ Life sciences ☐ Behavioural & social sciences ☐ Ecological, evolutionary & environmental sciences

For a reference copy of the document with all sections, see nature.com/documents/nr-reporting-summary-flat.pdf

Life sciences study design

All studies must disclose on these points even when the disclosure is negative.

Sample size	No sample size calculation was applied in this study to predetermine sample sizes for experiments using cell lines. A sample size of three was used as a starting point to evaluate the spread of the data (Casadevall A, Fang FC; Reproducible Science, Infect Immun, 2010 Dec; 78(12):4972-4975). Experiments were repeated more often if necessary to provide results with statistical significance.
Data exclusions	No data was excluded.
Replication	All experiments were replicated as stated in the figure legends.
Randomization	No randomization is applicable for flow cytometry as cells positive for Monobody binding are analysed.
Blinding	No blinding, as the same investigator performed most experiments and analyzed the data.

Reporting for specific materials, systems and methods

We require information from authors about some types of materials, experimental systems and methods used in many studies. Here, indicate whether each material, system or method listed is relevant to your study. If you are not sure if a list item applies to your research, read the appropriate section before selecting a response.

Materials & experimental systems

n/a	Involved in the study
☐	☒ Antibodies
☐	☒ Eukaryotic cell lines
☒	☐ Palaeontology and archaeology
☒	☐ Animals and other organisms
☒	☐ Clinical data
☒	☐ Dual use research of concern
☒	☐ Plants

Methods

n/a	Involved in the study
☒	☐ ChIP-seq
☐	☒ Flow cytometry
☒	☐ MRI-based neuroimaging

Antibodies

Antibodies used
mouse anti-V5 (Thermo Fisher Scientific, MA5-15253)
FITC-coupled anti-mouse IgG (Sigma-Aldrich, F0257-5ML)

Validation
All used antibodies were validated commercially. Certificates of analysis for the approved applications by the manufacturer are available on the company websites.

Eukaryotic cell lines

Policy information about cell lines and Sex and Gender in Research

Cell line source(s)
K562, DSMZ Cat# ACC-10, RRID:CVCL_0004

Authentication
cell lines from DSMZ were authenticated by the vendors (STR profiling and DNA barcoding).

Mycoplasma contamination
cell lines were regularly tested and were mycoplama negative

Commonly misidentified lines
(See ICLAC register)
no commonly misidentified cell lines were used in the study

Plants

Seed stocks
n/a

Novel plant genotypes
n/a

Authentication
n/a

Flow Cytometry

Plots

Confirm that:

☒ The axis labels state the marker and fluorochrome used (e.g. CD4-FITC).

☒ The axis scales are clearly visible. Include numbers along axes only for bottom left plot of group (a 'group' is an analysis of identical markers).

☒ All plots are contour plots with outliers or pseudocolor plots.

☒ A numerical value for number of cells or percentage (with statistics) is provided.

Methodology

Sample preparation
K562 cells were fixed in Paraformaldehyde 3.2 % for 10 min at room temperature and then permeabilized in >90% methanol for 20 min on ice. Monobody treatment was done as described in the Methods section.
EBY100 yeast cells for monobody selection and binding assays were treated and analysed as described in the Methods section.

Instrument	Guava easyCyte flow cytometer (Luminex)
Software	guavaSoft v4.0
Cell population abundance	K562 cell populations of Bcr-Abl-binding monobodies upon permeabilization were monitored using an intracellular streptavidin-AF488 detection. Abundance of cells binding the monobodies varied across experiments due to varying binding efficiencies.<br>Yeast cells displaying monobodies were detected using a mouse anti-V5 antibody and FITC-coupled anti-mouse IgG antibody. Abundance of yeast cells displaying the monobodies varied across experiments due to varying display efficiencies.
Gating strategy	Monobody binding was monitored based on streptavidin-AF488 detection.<br>Monobody display was monitored with a FITC-coupled anti-mouse IgG antibody.

☒ Tick this box to confirm that a figure exemplifying the gating strategy is provided in the Supplementary Information.
